# Long read sequencing reveals novel genomic and epigenomic alterations in repetitive regions of high grade serous ovarian cancer

**DOI:** 10.1038/s41598-025-21907-5

**Published:** 2025-10-30

**Authors:** Shiro Takamatsu, Jian Li, Thomas Welte, Eleonora Khlebus, Veena Vuttaradhi, Allison Brodsky, Barrett Lawson, R. Tyler Hillman

**Affiliations:** 1 Department of Gynecologic Oncology and Reproductive Medicine, The University of Texas MD Anderson Cancer Center, Houston, TX USA; 2 Department of Obstetrics and Gynecology, Kindai University Nara Hospital, Ikoma, Nara Japan; 3 Department of Anatomic Pathology, The University of Texas MD Anderson Cancer Center, Houston, TX USA; 4 Department of Obstetrics, Gynecology and Reproductive Sciences, Division of Gynecologic Oncology, Rebecca and John Moores Cancer Center, University of California, La Jolla, San Diego, CA USA; 5 San Diego Moores Cancer Center, University of California, 3855 Health Sciences Dr. La Jolla, San Diego, 92093 MC-0987, CA USA

**Keywords:** Cancer genomics, Ovarian cancer

## Abstract

Approximately half of high-grade serous ovarian carcinomas (HGSOCs) demonstrate homologous recombination deficiency (HRD) with characteristic genomic rearrangements. However, the impact of HRD on centromeres and transposable elements remains largely unexplored in HGSOC since conventional short-read sequencing is unable to interrogate these repetitive regions. We employed Oxford Nanopore long-read sequencing (LRS) to investigate genomic and epigenetic alterations in these regions. Pre-treatment archival cryopreserved tumor and matched blood samples were obtained for six patients with HGSOC. High-molecular-weight DNA was sequenced using Oxford Nanopore R10.4 flow cells and aligned to both the GRCh38 and the telomere-to-telomere T2T-CHM13 reference genome. Pathogenic gene mutations, allele-specific copy number variations, structural variants, and CpG methylation were analyzed. All six tumors had pathogenic *TP53* mutations. Two carried germline *BRCA1* mutations, while three showed *CCNE1* amplifications. HRD scores and mutational signatures associated with HRD were elevated in the *BRCA1*-mutated tumors. Centromeric regions were significantly hypomethylated in tumors and their methylation profiles distinctly separated HRD tumors from non-HRD tumors. LINE1 and ERV transposable elements showed marked hypomethylation in tumors without germline *BRCA1* mutations. Chromosome arm-specific telomere lengths were significantly shortened in tumors. Allele-specific hypermethylation in the *TERT* hypermethylated oncological region was detected in three tumors. LRS uncovered HRD-related genomic and epigenomic alterations in previously inaccessible repetitive regions of HGSOC, including centromeric and transposable element hypomethylation. These findings highlight the potential of such abnormalities as novel biomarkers for HGSOC and warrant further application of the methods to larger cohorts in future studies.

**Supplementary Information:**

The online version contains supplementary material available at 10.1038/s41598-025-21907-5.

## Introduction

Long-read sequencing (LRS) is a technology that enables the reading of significantly longer DNA sequences (10–100 kilobases) compared to conventional short-read sequencing (typically 50–250 nucleotides)^1^. Although sequencing accuracy was initially low at the time of LRS development, recent technological advancements have significantly improved its accuracy and reduced sequencing costs^2,3^. A major advantage of LRS is its ability to read long DNA strands directly without PCR amplification, allowing for high-resolution detection of large-scale insertions, deletions, complex structural variants (SVs), as well as repetitive sequences such as telomeres, centromeres, and interspersed nuclear elements^1^. Furthermore, LRS allows for high-accuracy haplotype phasing and the analysis of DNA methylation at the base level without requiring bisulfite treatment^4^. Utilizing these features, the Telomere-to-Telomere (T2T) consortium unraveled the previously unresolved genomic regions and successfully accomplished the first complete, gapless assembly of the human genome in 2022 (known as T2T-CHM13)^5^. The completion of this human reference genome has significantly contributed to rapid advancements in genetic disease diagnostics, population genetics, and evolutionary biology^1,6–8^. In cancer research, LRS is expected to enhance the detection of gene mutations and SVs, primarily by providing deeper insights into repetitive genomic elements that were largely inaccessible to conventional short-read sequencing platforms^4,9,10^.

High-grade serous ovarian carcinoma (HGSOC) is the most common histological subtype of ovarian cancer, accounting for approximately 70% of all cases^11^. Most cases are diagnosed at FIGO stage III–IV, exhibiting high recurrence rates and poor prognosis^11^. Nearly all HGSOC cases harbor *TP53* mutations and exhibit a high degree of chromosomal instability^12^. Additionally, *BRCA1* mutations are present in approximately 10–15% of cases, *BRCA2* mutations in 6–8%, and *BRCA1* promoter methylation in around 10%, with approximately 50% of cases demonstrating homologous recombination deficiency (HRD)^13,14^. Tumors with HRD have high sensitivity to platinum-based chemotherapy and PARP inhibitors^15^. On the other hand, *CCNE1* amplification, observed in approximately 25% of HGSOC cases^16^, is more common in non-HRD tumors and is associated with chemotherapy resistance and poor prognosis^17^.

In the early 2010s, genome-wide SNP array analyses revealed that HRD tumors exhibit a characteristic pattern of large-scale chromosomal structural rearrangements known as “genomic scars”^18^. This pattern has been evaluated by three different measures: Large-scale State Transitions (LST), Telomeric Allelic Imbalance (TAI) and Loss of Heterozygosity (LOH) scores^19^. The sum of these three scores, referred to as the HRD score, along with *BRCA1/2* mutation status, is currently used as a companion diagnostic HRD test for PARP inhibitor administration^20^. Recent advancements in whole-genome sequencing have identified distinct SV patterns between *BRCA1*-mutated and *BRCA2*-mutated HGSOC tumors^21,22^. It has been reported that non-HRD tumors harboring *CDK12* mutations exhibit unique SVs characterized by long tandem duplications, which are associated with particularly poor prognosis^23^. Additionally, subsets of prostate and ovarian cancers with *CDK12* loss-of-function mutations have shown increased neoantigen loads through gene fusions, suggesting that immune checkpoint inhibitors may serve as a promising therapeutic option^24^.

Comprehensive genomic analyses leveraging LRS and the novel T2T-CHM13 reference genome have not yet been reported for HGSOC. In order to determine the feasibility of this approach, we developed a pipeline for generating Oxford Nanopore R10.4.1 LRS from clinical HGSOC samples, aligning sequencing reads to both the conventional GRCh38 reference genome and novel T2T-CHM13 assembly, and performing downstream analyses of simple mutations and SVs. By applying this approach to a pilot cohort of HGSOC samples, we report novel genomic insights across previously inaccessible repetitive regions including the discovery of HRD-related mutational mechanisms affecting centromere structure.

## Methods

### Sample collection and DNA sequencing

Archival cryopreserved pre-treatment HGSOC samples with sufficient tumor purity > = 70% were selected based on expert (B.L.) pathologic evaluation. Genomic DNA was extracted from both tumor and matched normal blood samples. Libraries were prepared and sequenced on Oxford Nanopore PromethION R10.4.1 flow cells. Briefly, genomic DNA was sheared, end-repaired, ligated to sequencing adapters, and loaded onto flow cells. After sequencing, basecalling was performed using Dorado^25^. The resulting reads were then mapped to either the GRCh38 or T2T-CHM13 reference genomes using minimap2^26^.

### Simple mutation calling, allele-specific copy number analysis, and SV calling

Using sequencing data mapped to the GRCh38, germline and somatic short variants (SNVs/INDELs) were identified using established pipelines Clair3^27^ and ClairS^28^, respectively. Based on a previous study^29^, the following 29 genes were defined as related to homologous recombination repair: *ATM*,* ATR*,* BARD1*,* BLM*,* BRIP1*,* BRCA1*,* BRCA2*,* CDK12*,* CHEK1*,* CHEK2*,* FANCA*,* FANCC*,* FANCD2*,* FANCE*,* FANCF*,* FANCI*,* FANCL*,* FANCM*,* MRE11*,* NBN*,* PALB2*,* RAD50*,* RAD51*,* RAD51B*,* RAD51C*,* RAD51D*,* RAD52*,* RAD54L*,* RPA1*. Variants were annotated for their clinical significance, functional impact, and pathogenicity using Ensembl-VEP^30^, and those classified as “pathogenic” or “likely pathogenic” were retained.

Allele-specific copy number variations (CNVs), as well as tumor purity and ploidy, were inferred via a tumor–normal paired approach using ascatNGS^31^, and the HRD score (composed of LOH, TAI, and LST) was calculated using scarHRD^32^.

Previous studies have reported that integrating multiple somatic SV callers improves detection performance in cancer LRS data^33,34^. Accordingly, we employed four somatic SV callers, including Severus^35^, SAVANA^36^, nanomonsv^37^, and DELLY^38^, with default parameter settings for paired tumor-normal analysis. Variants shorter than 50 bp were excluded, and those identified by at least two of the four callers were retained as simple SVs. Complex SVs were identified using JaBbA^39^ with all detected SVs from the four callers as input. Single base substitutions (SBSs) and SV mutational signatures were calculated with the COSMIC signatures v3.4^40^ as a reference by non-negative least squares (NNLS).

### Centromeres and transposable elements (TEs)

Sequencing reads were mapped to the T2T-CHM13 reference genome, and genomic annotations for centromeres and satellite repeats were obtained from a T2T study^41^. Base-level read coverage was calculated using samtools^42^ and mosdepth^43^. The median read coverage for centromeric regions was computed per 1-kb genomic bin and normalized to the genome-wide median coverage for each sample.

Repeat sequences annotations of major TEs, including Long Interspersed Nuclear Element-1 (LINE1) Human-Specific subfamily (L1HS), Alu short interspersed nuclear element (SINE) subfamily Y (AluY), SINE-VNTR-Alu (SVA), and Endogenous RetroVirus (ERV) were obtained from a T2T study^44^. To analyze full-length of these insertions, we selected those with lengths > 6 kb for L1HS (*n* = 355), > 300 bp for AluY (*n* = 58288), > 1 kb for SVA (*n* = 2435), >1 kb for ERV (*n* = 271). The median read coverage was calculated per 1-kb for L1HS, SVA and ERV, and per insertion for AluY, normalized to the genome-wide median coverage for each sample.

### Quantification of 5-methylcytosine (5mC) levels in CpG sites, centromeres, and TEs

Sequence data mapped to the T2T-CHM13 reference genome with base modification information were processed using modkit^45^ to quantify 5mC levels across all genomic CpG sites. Methylation data for specific regions, including centromeric regions, inserted TE regions, and the gene loci of BRCA1, RAD51C, and TERT, were extracted using bedtools^46^. For centromeres and TE regions, CpG methylation levels were assessed as the average 5mC ratio (methylated reads / total reads) within each 1-kb genomic bin, while for AluY elements, the ratio was computed per insertion.

### Telomere length

Telomere length for each chromosome arm was estimated using Telometer^45^ and Telogator2^46^, based on reads mapped to the T2T-CHM13 genome supplemented with subtelomeric sequences. In addition, publicly available short-read whole genome sequencing data from the TCGA-OV study^16^ were obtained from the GDC portal^47^, and the average telomere length for each sample was estimated using TelSeq^48^.

### Supplementary methods

Detailed descriptions of the analysis workflow, codes, and software tools used in this study are provided in the Supplementary methods.

### Statistical analysis

This was a pilot study and no a priori power analysis was performed to guide sample size. All statistical analyses were performed using the Scipy package (v1.7.3) in Python (v3.8.8). Paired or unpaired t-tests were used to compare continuous variables between two groups. Levene’s test was used to compare variances between groups. P-values less than 0.05 were considered statistically significant.

### Ethical approval

Archival cryopreserved tumor tissue and blood of patients diagnosed with HGSOC were obtained from the M.D. Anderson Cancer Center Gynecologic Oncology Multidisciplinary Tumor Bank collected under an Institutional Review Board (IRB) approved collection protocol (#LAB02-188). Each patient provided written, informed consent for biospecimen collection and use for research applications. The use of these tumor bank samples for molecular sequencing analysis was also IRB-approved (#PA12-0305), and all procedures were performed in accordance with the relevant institutional and international guidelines and regulations.

## Results

### Investigation of non-repetitive regions

High-molecular-weight DNA was extracted from frozen, pre-treatment tumor tissues and matched blood samples from six patients with HGSOC (Supplementary Table 1) and sequenced using the Oxford Nanopore PromethION R10.4.1 platform. These LRS data were first mapped to the GRCh38 reference genome. The median coverage ranged from 25.48 to 39.77 (Supplementary Table 2).

A median of 7,621 somatic SNVs/INDELs were identified per tumor sample (range: 4,832 − 10,538) (Supplementary Fig. 1). Pathogenic *TP53* mutations were detected in all tumor samples and pathogenic germline *BRCA1* mutations were detected both in tumor and normal samples from P1 and P4 (Supplementary Table 1). No aberrant mutations were observed in other homologous recombination repair-related genes (Supplementary Table 3). No hypermethylation was detected in the promoter regions of *BRCA1* or *RAD51C* (Supplementary Fig. 2). COSMIC SBS mutational signature 3, reported to be associated with HRD^49^, was higher in the germline *BRCA1* mutated tumors T1 and T4 (t-test, *P* = .048; Table 1).

**Table 1:** Genomic profiles in the study cohort.

Variant		Sample identifiers					
		T1	T2	T3	T4	T5	T6
Somatic *TP53* mutation		+	+	+	+	+	+
Germline *BRCA1* mutation		+	-	-	+	-	-
*CCNE1* amp (major-minor copy number)		- (1–1)	+ (4 − 3)	- (2–2)	- (2 − 1)	+ (7 − 2)	+ (6 − 2)
Tumor ploidy		1.8	4.57	3.56	3.09	2.61	3.26
Tumor purity (%)		77	35	39	81	71	90
Simple SVs	Deletion	101	84	56	272	92	26
	Duplication	65	19	114	109	24	74
	Translocation	85	18	18	115	91	27
	Insertion	9	18	1	9	8	1
	Inversion	14	37	6	46	22	16
Complex SV	Chromoplexy	3	3	0	1	1	1
	Chromothripsis	0	1	0	0	0	0
	Pyrgo	0	0	16	1	0	10
	Breakage-fusion-bridge	0	0	0	1	0	1
	Double minutes	0	0	0	0	0	1
	Templated insertion chains	4	0	0	5	2	0
	Rigma	0	2	2	10	2	2
HRD score		70	65	34	88	52	26
LST score		26	24	4	42	18	7
LOH score		20	13	10	17	9	6
TAI score		24	28	20	29	25	13
COSMIC SBS 3 (%)		16.3	0.0	9.2	13.9	8.1	0.0
COSMIC SV 3 (%)		27.5	0.0	1.8	25.6	0.8	4.2

SV; Structural Variants, HRD; Homologous Recombination Deficiency, LST; arge-scale State Transitions, LOH; Loss of Heterozygosity, TAI; Telomeric Allelic Imbalance, SBS; Single Base Substitution.

Allele-specific CNV analysis using ascatNgs^31^ (Supplementary Fig. 3) revealed *CCNE1* amplification, defined as a total copy number six or more, in three tumors without germline *BRCA1* mutations (T2, T5, and T6) (Table 1). These *CCNE1*-amplified tumors were associated with shorter progression-free and overall survival (Supplementary Table 1). The HRD score tended to be higher in tumors with germline *BRCA1* mutations (T1 and T4) compared to the other four tumors (t-test, *P* = .072; Table 1).

To improve SV detection accuracy, we integrated results from four somatic SV callers for LRS—Severus^35^, SAVANA^36^, nanomonsv^37^, and DELLY^38^—using default settings. Variants shorter than 50 bp were excluded, and those supported by at least two callers were retained as simple SVs (Table 1, Supplementary Fig. 4, see Methods). The contribution of COSMIC SV3 signature, reported to be linked to HRD^49^, was higher in germline *BRCA1*-mutated tumors T1 and T4 (t-test, *P* = .027; Table 1).

For complex SVs, chromoplexy, defined as a chain of interdependent rearrangements involving multiple chromosomes in a single event^39^, was observed in five of six tumors (Table 1, Supplementary Fig. 5). Templated insertion chains, characterized by serial insertions of short DNA segments derived from distant genomic loci and associated with BRCA deficiency^50^, were frequently detected in the germline *BRCA1*-mutated tumors T1 and T4 (Table 1). Pyrgo, representing towers of low-junction copy number duplications found in ovarian and breast cancers^39^, was enriched in T3 and T6 (Table 1). Large-scale rearrangements indicative of chromothripsis, a catastrophic form of chromosomal shattering and reassembly^39^, was observed in T2 (Supplementary Fig. 5). High-level focal amplifications suggesting extrachromosomal DNA^39^ and breakage-fusion-bridge cycles^39^ were detected in T6 (Supplementary Fig. 5). These SVs are consistent with the previously reported complex SV profiles in ovarian cancer^51,52^.

Taken together, these results demonstrate the feasibility of LRS in detecting gene mutations and structural alterations in non-repetitive genomic regions as an approach comparable to conventional short-read methods for characterizing HGSOC.

### Epigenetic alterations in centromere regions

**Fig. 1:**
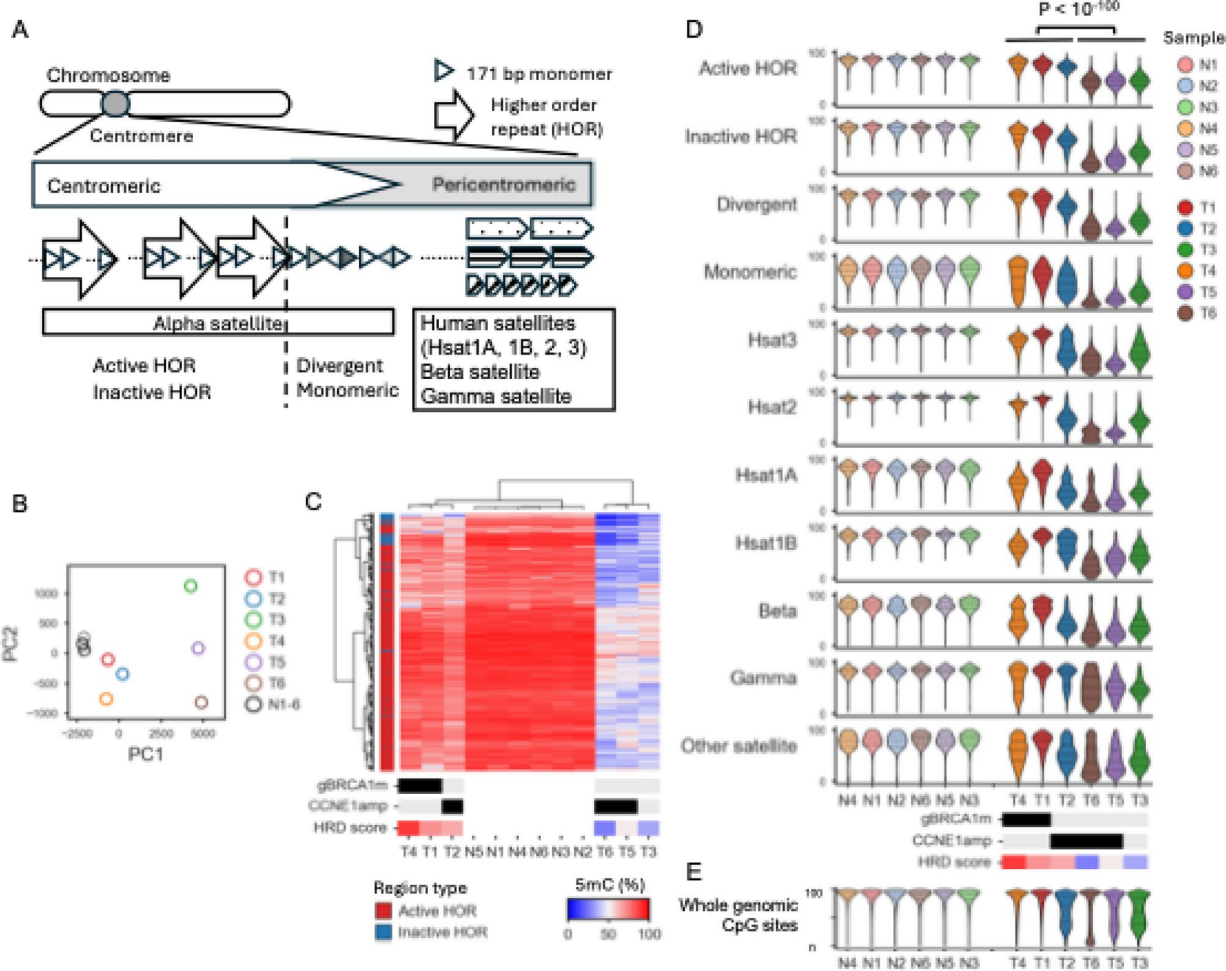
Epigenomic alterations in centromere regions. (**A**) Schematic of satellite DNA structure in the human centromere. Alpha satellites consist of 171 bp monomers arranged into higher-order repeats (HORs), classified as active, inactive, divergent, and monomeric satellites. Pericentromeric regions contain satellite families including HSat1 (A, B), HSat2, HSat3, beta, gamma, and other satellites. (**B**) Principal component analysis (PCA) based on CpG methylation levels per 1-kb window in active and inactive HOR regions. All six normal samples (N1-N6, black circles) clustered closely in a single region. In contrast, tumor samples (T1-T6, color circles) were broadly dispersed away from the normal cluster, highlighting a marked difference in methylation status between tumor and normal samples. Notably, T1, T2, and T4 clustered together along the first principal component, as did T3, T5, and T6; however, the latter group exhibited greater dispersion along the second principal component. (**C**) Unsupervised hierarchical clustering based on CpG methylation levels per 1-kb window in active and inactive HOR regions. Rows represent 1-kb genomic bins, totaling 16,851 for active HOR regions and 1573 for inactive HOR regions, while columns correspond to individual samples. Unsupervised hierarchical clustering was based on Ward’s method. Tumor samples were separated into two distinct groups, corresponding to high HRD score (T4, T1, T2) and low HRD score (T6, T5, T3), as shown in the color bar below the heatmap. gBRCA1m: presence of BRCA1 mutation; CCNE1 amp: presence of CCNE1 amplification. Region type (active vs. inactive HOR) is indicated on the bar left of the heatmap. Methylation levels are represented by a color gradient from blue (low) to red (high). (**D**) CpG methylation status in various satellite types across normal (N) and tumor (T) samples. Samples are ordered consistently with the clustering result of tumor samples shown in panel C. Each violin plot shows the distribution of average CpG methylation levels per 1-kb bin within a given satellite type. Overall, tumor samples showed markedly lower CpG methylation compared to normal blood controls across all satellite types. Among tumor samples, low-HRD score group (T6, T5, T3) showed stronger hypomethylation than high-HRD score group (T4, T1, T2). Differences between the two tumor groups were significant for all satellite types (*P* < 10⁻¹⁰⁰, t-test). (**E**) CpG methylation status in whole genomic CpG sites. Tumor samples showed moderate and variable levels of hypomethylation, a pattern commonly referred to as global DNA hypomethylation in cancer [59].

The centromere is a distinct chromosomal region composed of highly repetitive DNA sequences that plays an important role in accurate chromosome segregation during mitosis^53^. The central part of the human centromere primarily consists of α-satellite DNA, characterized by a multi-layered tandem repeat structure based on a 171 bp monomer as the smallest unit^53^. Typically, 2 to 34 monomers are arranged in tandem to form a higher-order repeat (HOR), and these HORs are further repeated in tandem hundreds to thousands of times, forming a highly organized chromatin structure essential for kinetochore formation^53^ (Fig. 1A). The T2T Consortium recently reported comprehensive genomic and epigenomic maps of the human centromere, providing detailed annotations of repetitive regions^41^. They categorized α-satellites into active or inactive HORs, monomeric, and divergent satellites, along with other satellite repeats, which are typically located in pericentromeric regions, into human satellite families (Hsat1A, Hsat1B, Hsat2, and Hsat3), beta satellites, and gamma satellites (Fig. 1A). According to these annotations, sequencing reads mapped to the T2T-CHM13 reference genome were partitioned into 1-kb bins within centromeric regions, and the average read coverage and CpG methylation levels were analyzed (see Methods).

Read coverage varied widely across satellite types and chromosomes (Supplementary Fig. 6A). Exceptionally high coverage was observed in the active HOR region of chromosome 21 (Supplementary Fig. 6B) and low coverage in the active HOR region of chromosome 6 (Supplementary Fig. 6C). Five of six tumor samples showed significantly lower read coverage compared to matched normal samples in α-satellite regions (Supplementary Fig. 6D), which is consistent with previous PCR-based studies that reported highly variable and quantitatively reduced copy numbers of centromeric α-satellite arrays in cancer cells^54,55^.

Nanopore sequencing has been shown to provide high-resolution and reliable 5mC methylation analysis even at low coverage (less than 10x)^56,57^. In our LRS data, we observed no correlation between read coverage and methylation level at CpG sites both across chromosomes and in centromeres (Supplementary Fig. 7A). Furthermore, differentially methylated CpG sites between HGSOCs and white blood cells extracted from publicly available methylation array datasets reproduced similar methylation patterns in our LRS samples (Supplementary Fig. 7B), confirming the validity of our LRS-derived methylation profiles.

CpG methylation profiles in active and inactive HOR regions were calculated as average 5mC ratios per 1-kb bin (see Methods), and the resulting bin-wise methylation data were used as input features for principal component analysis (PCA). The PCA plot revealed that all six normal samples clustered closely in a single region of the PCA space, whereas tumor samples were broadly dispersed away from the normal cluster, indicating a marked difference in methylation status between tumor and normal samples in these regions (Fig. 1B). Furthermore, unsupervised hierarchical clustering using Ward’s method based on the same centromeric CpG methylation profiles separated tumor samples into two groups: one comprising T1, T2, and T4 (left) and the other comprising T3, T5, and T6 (right) (Fig. 1C). Previous studies^29,58^ reported an HRD score threshold of 63 as an optimal cutoff for identifying HRD-positive tumors within HGSOC. Based on this criterion, we classified T1, T2, and T4 as the high-HRD score group, and T3, T5, and T6 as the low-HRD score group (Fig. 1C).

In all types of centromeric regions, tumor samples exhibited significantly lower CpG methylation levels than normal blood samples (Fig. 1D). Among tumors, those classified as low-HRD score group (T3, T5, T6) showed significantly more pronounced hypomethylation than high-HRD score group (T1, T2, T4) (t-test, *P* < 10^− 100^ for all comparisons, Fig. 1D). For comparison, we also examined the average methylation levels of genome-wide CpG sites (Fig. 1E), where tumor samples showed moderate and variable levels of hypomethylation, a pattern commonly referred to as global DNA hypomethylation in cancer^59^. While hypomethylation patterns observed in centromeric satellite regions partially mirrored this global trend, they were consistently more hypomethylated and showed distinct regional patterns. These findings suggest that hypomethylation in centromeric regions may be partly driven by global methylation changes but likely reflects additional region-specific epigenomic regulatory mechanisms.

Considering the reported association between centromeric methylation and chromosomal instability^60^ and the crucial roles of *BRCA1* and homologous recombination repair in maintaining centromere stability^61–64^, these observations suggest that epigenetic alterations in centromeric regions may be associated with the HRD status of tumors.

### Increased read coverage variance and CpG methylation in TEs

**Fig. 2:**
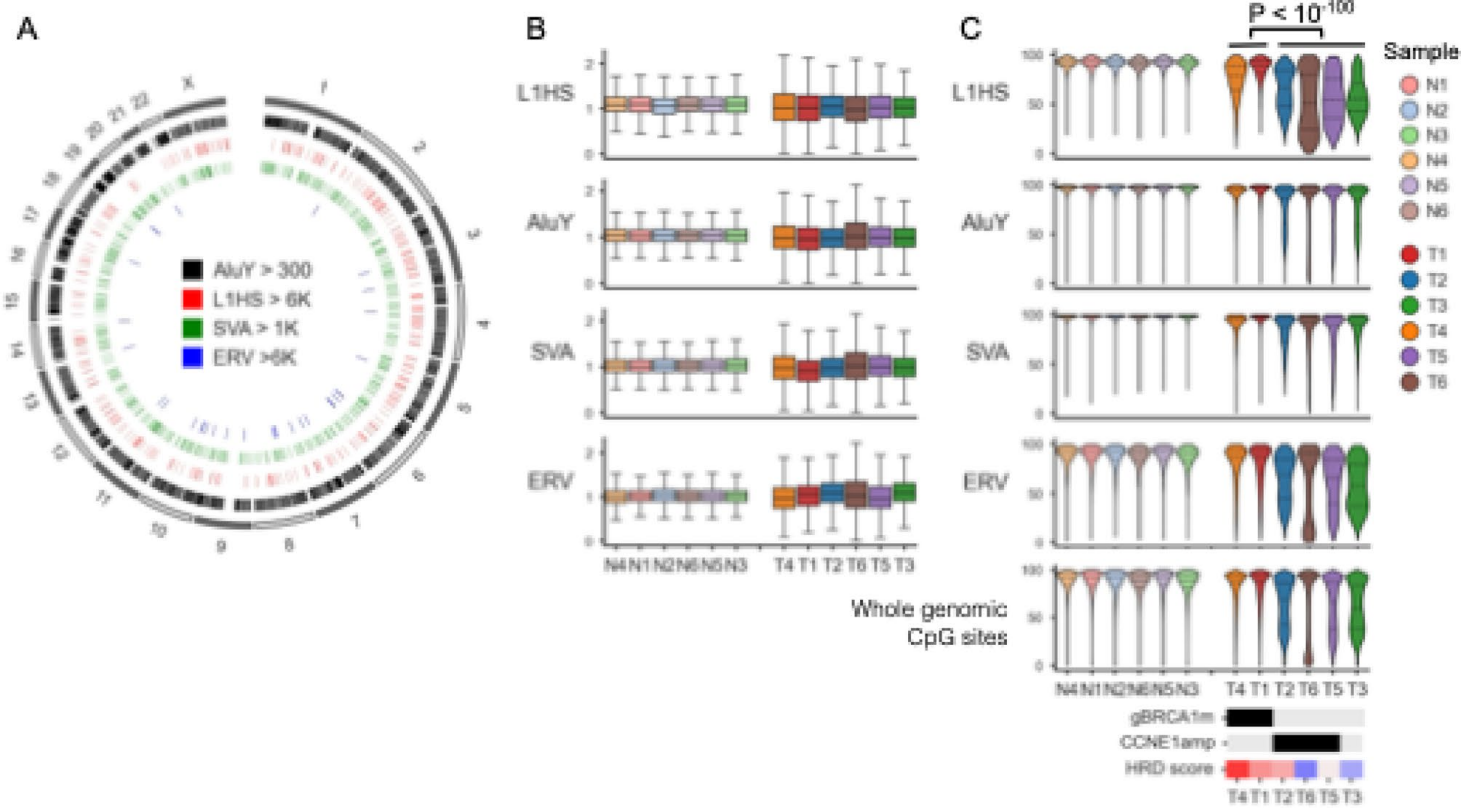
Read mapping coverage and CpG methylation in transposable elements (TEs) . (**A**) Genomic distribution of known TE insertions. To analyze full-length TEs, we extracted L1HS ( > = 6 kb), AluY ( > = 300 bp), SVA ( > = 1 kb), ERV ( > = 1 kb) using repeat annotations based on a previous T2T study^44^. Circos plot shows their chromosomal locations across the genome. (**B**) Normalized median read coverage of TEs in normal (N) and tumor (T) samples. For each TE class, median coverage was calculated per 1-kb region (or per insertion for AluY), normalized to the gnome-wide median coverage in each sample. Samples are ordered consistently with the clustering result shown in Fig. 1C. While the median coverage was similar between normal and tumor samples, the variance was significantly higher in tumor samples across all TE types. (**C**) CpG methylation levels of TEs and whole-genome CpG sites in normal (N) and tumor (T) samples. Methylation level was measured as the proportion of 5mC per 1-kb window (or per insertion for AluY). Bottom panel shows the global CpG methylation for comparison (identical to Fig. 1E). Compared to the genome-wide global hypomethylation, L1HS and ERV elements showed more pronounced hypomethylation, while AluY and SVA showed milder changes. Among tumors, those without *BRCA1* mutations (T2, T6, T5, and T3) showed stronger hypomethylation (t-test, *P* < 10^–100^ for all comparisons).

TEs are mobile, repetitive DNA sequences that comprise approximately 45% of the human genome^44,65^. In normal cells, TEs are primarily silenced through DNA methylation. However, in cancer cells, certain types of TEs, including long interspersed nuclear element-1 (LINE1) and human endogenous retroviruses (ERVs), can be reactivated due to DNA demethylation, contributing to tumor development and genomic instability^66,67^. Their repetitive nature has made accurate analysis challenging with conventional short-read sequencing platforms. T2T Consortium recently addressed this by utilizing LRS to produce the complete T2T-CHM13 assembly, which includes the reference locations of these elements^44^. Using the T2T-CHM13 aligned sequencing data and the reported repeat annotations, we analyzed major types of TEs, including LINE1 human-specific subfamily (L1HS), Alu short interspersed nuclear element (SINE) subfamily Y (AluY), SINE-VNTR-Alu (SVA), and ERV (Fig. 2A).

Although the median coverage of these elements did not differ between tumor and normal samples, their variance was significantly higher in tumor samples across all elements (Fig. 2B) (Levine test, *P* < 2.1 × 10^− 6^ for all comparisons). A recent genome-wide analysis demonstrated that the number of TE repeats vary substantially in association with chromosomal instability in cancer^68^. Thus, the observed increase in coverage variance may reflect the high level of chromosomal instability characteristic of HGSOC.

As with the centromeric regions, CpG methylation levels in TEs were calculated as the average 5mC ratios per 1-kb bin for each TE type (or per insertion for AluY). All TE types showed significant hypomethylation in tumor samples compared to normal blood samples (Fig. 2C). Compared to the genome-wide global hypomethylation in tumors, L1HS and ERV elements showed more pronounced hypomethylation, while AluY and SVA showed milder changes (Fig. 2C). These varying degrees of hypomethylation among TE types may reflect differences in the extent to which epigenetic de-repression of these elements provides selective advantages for tumorigenesis. Among tumor samples, those without *BRCA1* mutations showed stronger hypomethylation (t-test, *P* < 10^− 100^ for all comparisons, Fig. 2C), suggesting the extent of global hypomethylation may vary with the molecular characteristics of the tumor.

### Telomere shortening and TERT hypermethylated oncological region (THOR) hypermethylation

**Fig. 3:**
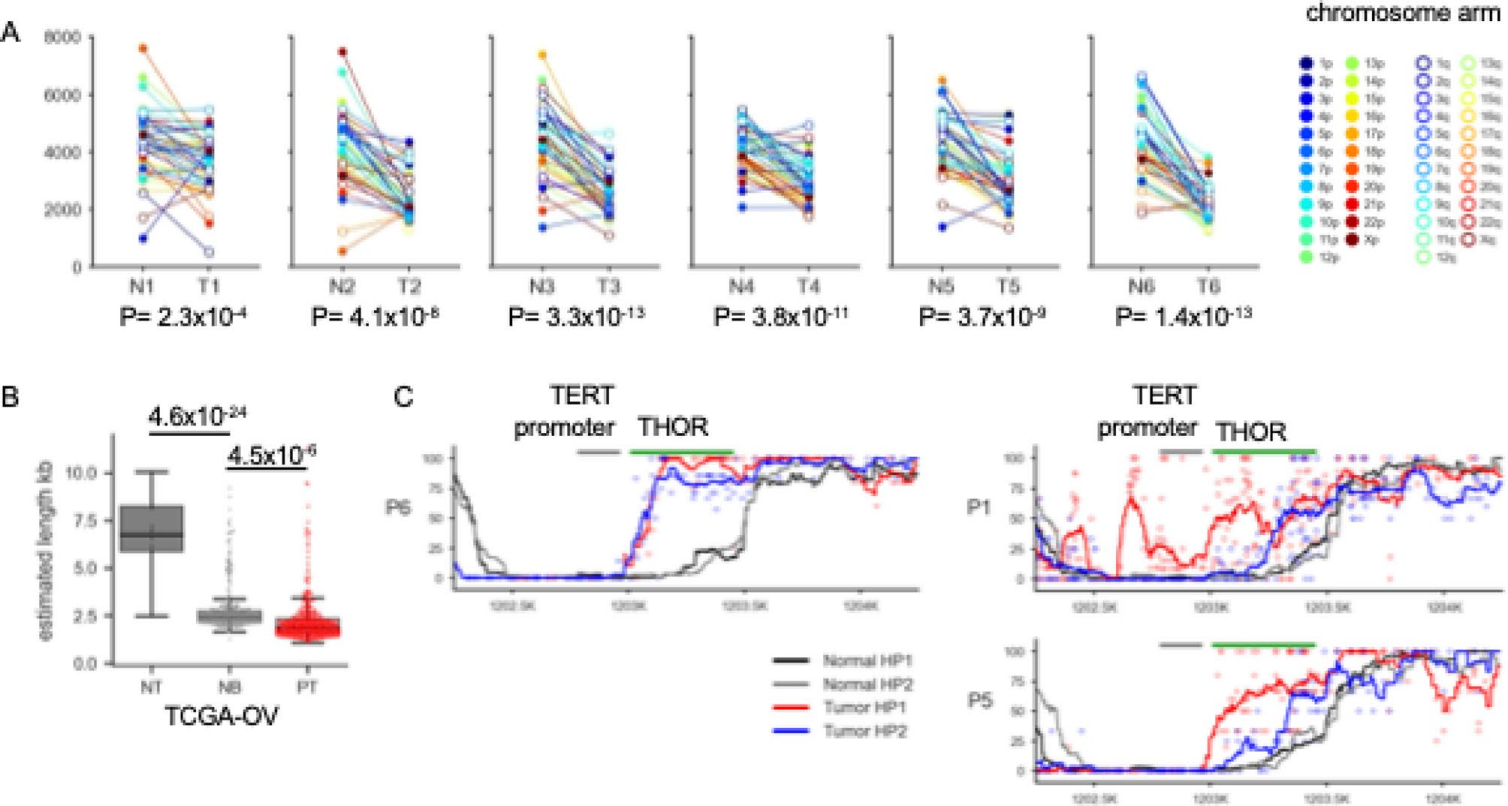
Telomere length shortening and* TERT* hypermethylated oncological region (THOR). (**A**) Estimated telomere length per chromosomal arm in matched normal (N) and tumor (T) samples. Each point represents a specific chromosome arm, with lines connecting corresponding arms in paired samples. The estimated telomere length was significantly shorter in tumor samples than in matched normal samples in all cases. P-values were calculated by paired t-test. (**B**) The average telomere length estimated from short-read whole genome sequencing in TCGA-OV samples. The average telomere length was significantly shorter in the order of non-cancerous tissue (NT), normal blood cells (NB), and ovarian cancer tissue (PT). P-values were calculated by unpaired t-test. (**C**) Allele-specific methylation status of the TERT promoter and THOR region in three tumor samples. Biallelic THOR hypermethylation was observed in sample T6, while monoallelic THOR hypermethylation was observed in T1 and T5. CpG methylation levels (y-axis) across the genomic region (x-axis, T2T-CHM13 coordinates) are shown for normal and tumor haplotypes. Individual CpG site methylation levels are indicated by dots (black/grey: normal haplotypes, red/blue: tumor haplotypes), with smoothed averages values represented by lines calculated using a 100-bp sliding window. CpG sites with fewer than 3 supporting reads were excluded.

Recent advances in LRS technologies have enabled high-resolution analysis of telomere repeat sequences at the chromosome arm level^45^. We calculated telomere length for each chromosome arm using Telometer^45^. Most telomeres were significantly shorter in cancer samples than in matched blood samples (Fig. 3A, Supplementary Fig. 9). This result was reproduced when using an alternative tool, Telogator2^46^ (Supplementary Fig. 10).

Previous studies have shown that blood cells typically exhibit the shortest telomeres among normal tissues^69^. Consistent with this, our analysis of TCGA-OV whole-genome sequencing data revealed a significant decreasing trend in average telomere length of all chromosomes, in the order of non-cancerous tissue, normal blood cells, and HGSOCs (Fig. 3B). This result supports significant shortening of telomeres in HGSOC.

Recent studies have shown that hypermethylation of the *TERT* hypermethylated oncological region (THOR) is associated with telomerase activation in multiple cancers, including ovarian cancer^70^, and that THOR methylation occurs in an allele-specific manner^71^. In our dataset, one tumor (T6) showed biallelic hypermethylation of the THOR region, while two tumors (T1 and T5) displayed monoallelic hypermethylation (Supplementary Fig. 11A), which was possibly difficult to detect without haplotype-specific methylation analysis, a method uniquely enabled by LRS (Supplementary Fig. 11B). These results suggest that THOR hypermethylation is one of the mechanisms of telomerase activation in HGSOC that has been underestimated in conventional approaches.

## Discussion

This study is the first report performing LRS on clinical primary tumor samples for HGSOC. Recently, a large-scale cohort study employing LRS on advanced cancer patients was reported; however, it included only three metastatic samples of HGSOC, using an older-generation Nanopore sequencing platform, R9.4.1^72^. In the present study, we firstly applied allele-specific CNV analysis and HRD score calculation on nanopore LRS data derived from clinical HGSOC samples. Furthermore, to investigate repetitive genomic regions, we employed a novel approach by mapping reads to the T2T-CHM13 reference genome. This analysis pipeline and the resulting data constitute valuable resources for future research.

Even in this small pilot cohort, novel genomic mechanisms of HGSOC were uncovered using our approach. Centromeres contain α-satellite repetitive DNA sequences essential for accurate chromosome segregation during cell division, typically maintained in a highly methylated heterochromatin state. In cancer cells, abnormal hypomethylation in these regions can disturb the localization of centromere-specific protein CENP-A and impair kinetochore function, leading to chromosome instability and tumor progression^60,64^. Our study identified significant hypomethylation in centromere regions of HGSOC samples compared to normal blood controls (Fig. 1D). Although normal ovarian or fallopian tube epithelium were not included as controls, and thus we cannot exclude the possibility that the observed differences reflect tissue-specific methylation patterns, a previous study reported that DNA methylation of LINE-1 and Alu elements is lower in epithelial ovarian cancer than in white blood cell, normal ovarian surface epithelium, and fallopian tube epithelium^73^. The centromeric hypomethylation observed in tumor samples may account for the high levels of genomic instability and aneuploidy characteristic of this disease. Notably, we found that centromeric hypomethylation patterns differed by HRD status (Fig. 1C). Under normal conditions, centromeres are transcriptionally active and produce non-coding RNAs via RNA polymerase II, leading to the formation of DNA: RNA hybrid structures known as R-loops^74^. These R-loops can stall and collapse replication forks, causing replication stress, DNA damage, and chromosomal instability^74^. Recent studies have shown that *BRCA1* localizes to centromeres during the G1 phase to resolve the R-loops and maintain centromere stability^61^, and that the RAD51 complex is similarly recruited to centromeres in G1 through R-loops, CENP-A and related factors to repair double-strand breaks via homologous recombination^62^. Collectively, recent studies highlight that homologous recombination repair plays a critical role not only in high-fidelity DNA repair during the G2/M phase but also in resolving R-loops at centromeres during the G1 phase; dysfunction of this process leads to centromeric instability and elevated replication stress. Therefore, the intertumoral differences in centromeric hypomethylation patterns observed in the present study may be associated with variations in HRD status, levels of aneuploidy, and replication stress. If so, genomic and epigenomic alterations in centromeric regions may provide additional information to help refine patient stratification beyond conventional HRD scoring or *BRCA1/2* mutation status. Although still in its early stages, this study suggests that LRS-based analysis of centromeric regions may lead to the identification of new biomarkers that support more precise treatment decisions, particularly in atypical HGSOC subgroups where existing predictive markers are insufficient.

In this study, we observed pronounced hypomethylation of LINE1/ERV elements in tumor samples compared to genome-wide CpG sites or AluY/SVA elements (Fig. 2C, D). These TEs differ in their autonomous activities. Specifically, LINE1 contains ORF1 and ORF2 genes that encode reverse transcriptase and endonuclease, which are essential for autonomous retro-transposition, whereas ERVs have long terminal repeats (LTRs) with strong promoter activity and partial coding potential for envelope proteins. Many previous studies have demonstrated that demethylation of LINE1 and ERV elements leads to aberrantly increased transcription and transposition activity, contributing to tumor development and genomic instability in cancer^65^. In contrast, SINE/AluY and SVA elements lack autonomous transpositional activity; thus, their hypomethylation in cancer is less frequently reported and may not necessarily confer a selective advantage for tumor progression. These differences in selective pressure for hypomethylation likely explain the observed results. Furthermore, *TP53* is known to directly suppress LINE1 activity^75^, and loss of p53 function can induce increased LINE1 expression and retro-transposition, which promote genomic instability and tumor progression^76^. Previous studies have reported that hypomethylation and overexpression of the LINE1 promoter occur in 100% (12/12) of HGSOC and in 78% (14/18) of its precursor lesion, serous tubal intraepithelial carcinoma (STIC)^77^, supporting our findings.

Telomere maintenance mechanisms are essential for cancer cells to avoid cellular senescence and apoptosis, allowing for unlimited proliferation, a hallmark of cancer^78^. Although over 95% of cancers activate telomerase via *TERT* promoter mutations, amplifications, or alternative telomere lengthening (ALT) mechanisms^79^, these alterations are not typically observed in HGSOCs^80–82^. Here, we observed allele-specific methylation of the previously reported *TERT* hypermethylated oncological region (THOR)^70,71^ in three of six HGSOC cases (Fig. 3C). Given that THOR methylation occurs in an allele-specific manner, its detection can be challenging without the haplotype phasing capability afforded by LRS (Supplementary Fig. 11) and thus has been underestimated previously. THOR methylation may represent a telomerase activation mechanism in HGSOC and warrants further investigation. In addition, this study is the first to use LRS to analyze allele-specific THOR methylation in cancer, highlighting the potential for broad application in future epigenetic studies.

This study has several key limitations. Firstly, we used the T2T-CHM13 reference genome, derived from a single complete hydatidiform mole cell line^5^, which is nearly fully homozygous, whereas typical human genomes are heterozygous dipolid. Repetitive regions are highly variable both among individuals and between the two haplotypes within a single individual. The exceptionally high or low read coverage observed in specific chromosomes (Supplementary Fig. 6) likely reflects structural differences between the reference and our clinical samples. Secondly, the centromeric dip region, which has been previously reported to be characterized by prominent CpG hypomethylation within the active HOR region and is critical for CENP-A localization, kinetochore assembly and centromere function^41^, was not clearly identified (Supplementary Fig. 8). Althghoug LRS data theoretically enables haplotype-resolved analysis, factors such as mixed somatic mutations, copy number variations, tumor purity, and intratumoral heterogeneity in clinical samples hinder complete phasing, especially in regions with low read coverage. Thirdly, we used white blood cells as the normal controls for comparison with tumor samples. Because the DNA methylation profiles of white blood cells and normal epithelial cells differ substantially^83,84^, observed differences between normal and tumor samples may reflect inherent differences between cell types rather than tumor-specific alterations. Finally, the small sample size limits the generalizability of our findings; larger studies will be needed to validate the genomic alterations detected here.

In conclusion, this study demonstrates the utility of LRS in elucidating the complex genomic and epigenetic landscapes of HGSOC, highlighting potential biomarkers and mechanisms underlying genomic instability in these tumors. The end-to-end pipeline reported here leverages LRS and the novel T2T-CHM13 reference genome, supporting larger future investigation of repetitive genomic elements critical to the pathogenesis of HGSOC.

## Supplementary Information

Below is the link to the electronic supplementary material.

Supplementary Material 1

Supplementary Material 2

## Data Availability

DNA sequencing data related to this study have been deposited with the European Genome-phenome Archive (EGAD50001509). All data requests will be granted by the Data Access Committee. Controlled-access data from the TCGA ovarian cancer study are available through dbGaP study accession number phs000178.
