# Supplementary material for: Long read sequencing reveals novel genomic and epigenomic alterations in repetitive regions of high grade serous ovarian cancer
Source: Sci Rep. 2025 Oct 30;15:38028. doi: 10.1038/s41598-025-21907-5 (PMC12575789; doi:10.1038/s41598-025-21907-5)
Supplement: Supplementary file 1 — Supplementary Material 1 [file 41598_2025_21907_MOESM1_ESM.pdf]

Supplementary data

Supplementary Figures

Supplementary Figure 1: Distribution of variant allele frequency of detected somatic SNV/INDELs using ClairS

Supplementary Figure 2: No promoter hypermethylation was observed in BRCA1 or RAD51C genes

Supplementary Figure 3: Allele-specific copy number analysis using ascatNGS

Supplementary Figure 4: Overlaps of detected structural variants between four somatic callers

Supplementary Figure 5: Complex structural variants detected using JaBbA

Supplementary Figure 6: Read coverage of centromeric regions

Supplementary Figure 7: Validation of methylation profiles of the studied samples

Supplementary Figure 8: Detailed CpG methylation analysis for each chromosome centromere

Supplementary Figure 9: Estimated telomere length per chromosome arm calculated by Telometer

Supplementary Figure 10: Telomere length shortening in tumor samples analyzed by Telogator2

Supplementary Figure 11: Allele-specific and non-specific THOR hypermethylation

Supplementary Tables

Supplementary Table 1: Patient characteristics

Supplementary Table 2: Quality control metrics for Oxford Nanopore long-read sequencing

Supplementary Table 3: Pathogenic somatic mutation profile (provided in a separate Excel file)

Supplementary Methods

Analysis pipeline overview

Sample collection, preparation, and long-read whole genome sequencing

Detection of pathogenic SNVs/INDELs

Allele-specific copy number variation, tumor purity, ploidy and the HRD score

Detection of somatic structural variants (SVs)

Centromeres and Transposable elements (TEs)

Quantification of 5-methylcytosine (5mC) levels in CpG sites, centromeres, and TEs

Telomere length

Validation of methylation profiles of the studied samples using external data

Software versions

Supplementary References

Supplementary Figures

Supplementary Figure 1: Distribution of variant allele frequency of detected somatic SNV/INDELs using ClairS [1]

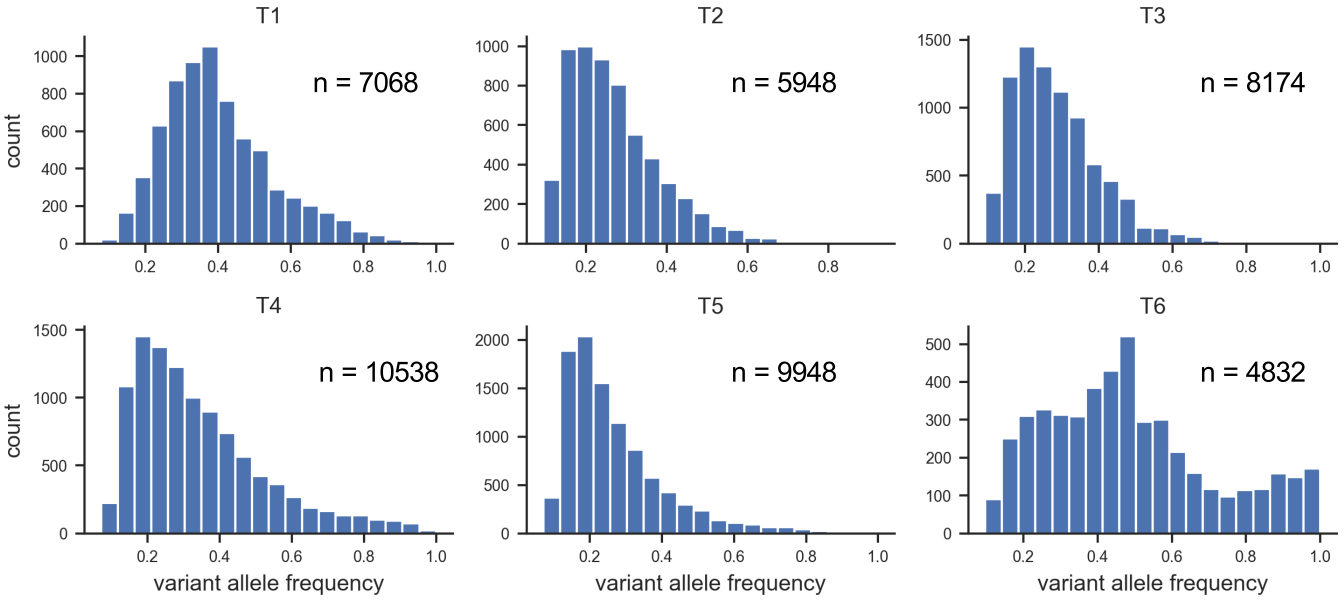

Histograms show the frequency distribution of variant allele frequencies (VAFs) for somatic SNVs and INDELs identified in six tumor samples (T1–T6). The number of variants analyzed in each sample is indicated (n). Most samples exhibited a unimodal distribution peaking at low to intermediate VAF values (~0.2–0.4), whereas sample T6 showed a broader and more heterogeneous distribution pattern, suggesting potential subclonal complexity.

Supplementary Figure 2: No promoter hypermethylation was observed in BRCA1 or RAD51C genes

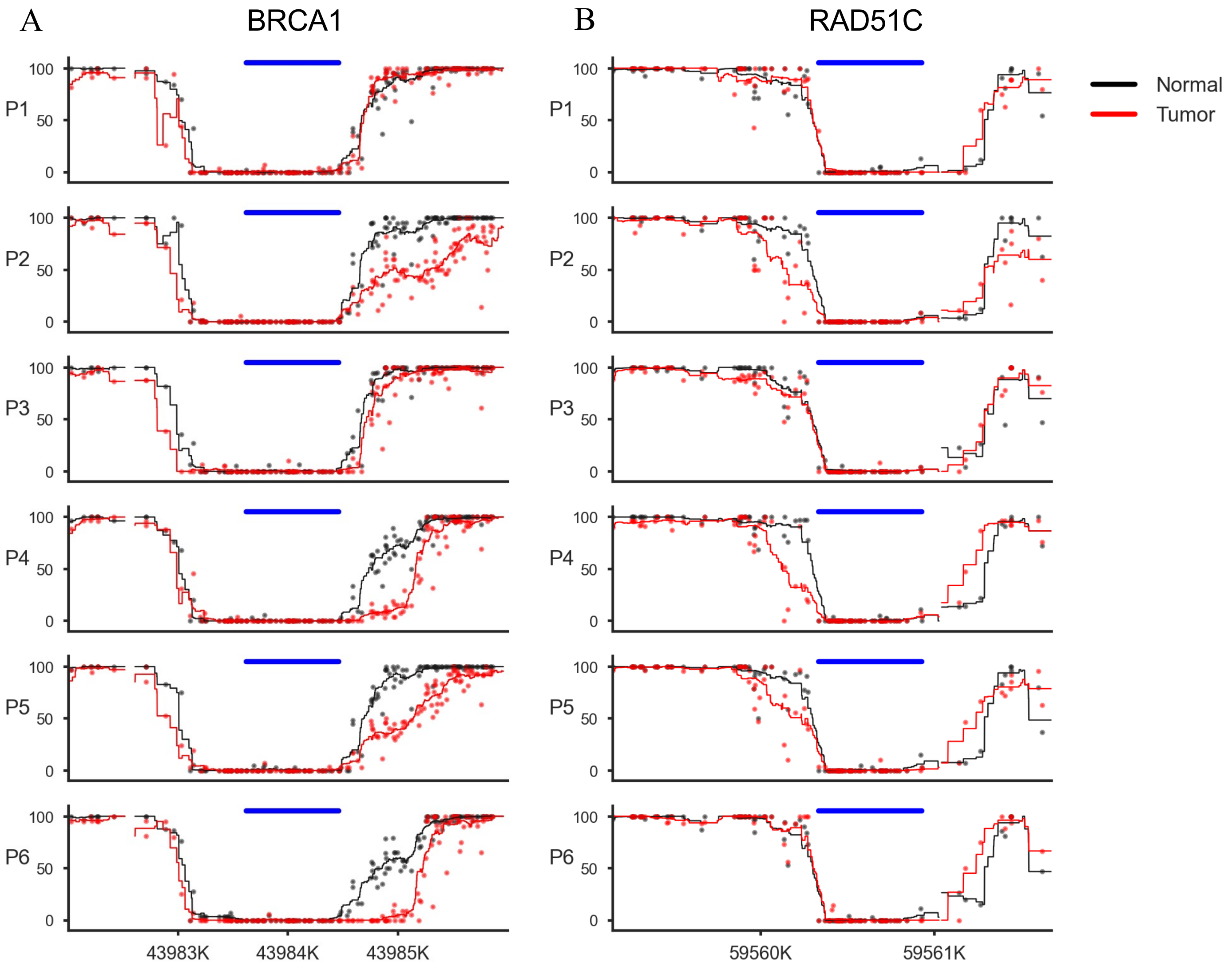

DNA methylation of BRCA1(A) and RAD51C (B) gene promoter regions in tumor and normal samples. Each panel represents an individual patient (P1–P6). The x-axis indicates genomic coordinates (in kilobases), and the y-axis represents CpG methylation levels (%). Black and red dots indicate methylation rates of individual CpG sites in normal blood and tumor tissue DNA, respectively. CpG sites with fewer than 5 reads were excluded. Black and red lines represent smoothed methylation levels calculated as moving averages using a 200-bp sliding window. The blue horizontal bar indicates the promoter CpG island regions previously reported to be associated with transcriptional silencing due to hypermethylation [2].

Supplementary Figure 3: Allele-specific copy number analysis using ascatNGS [3]

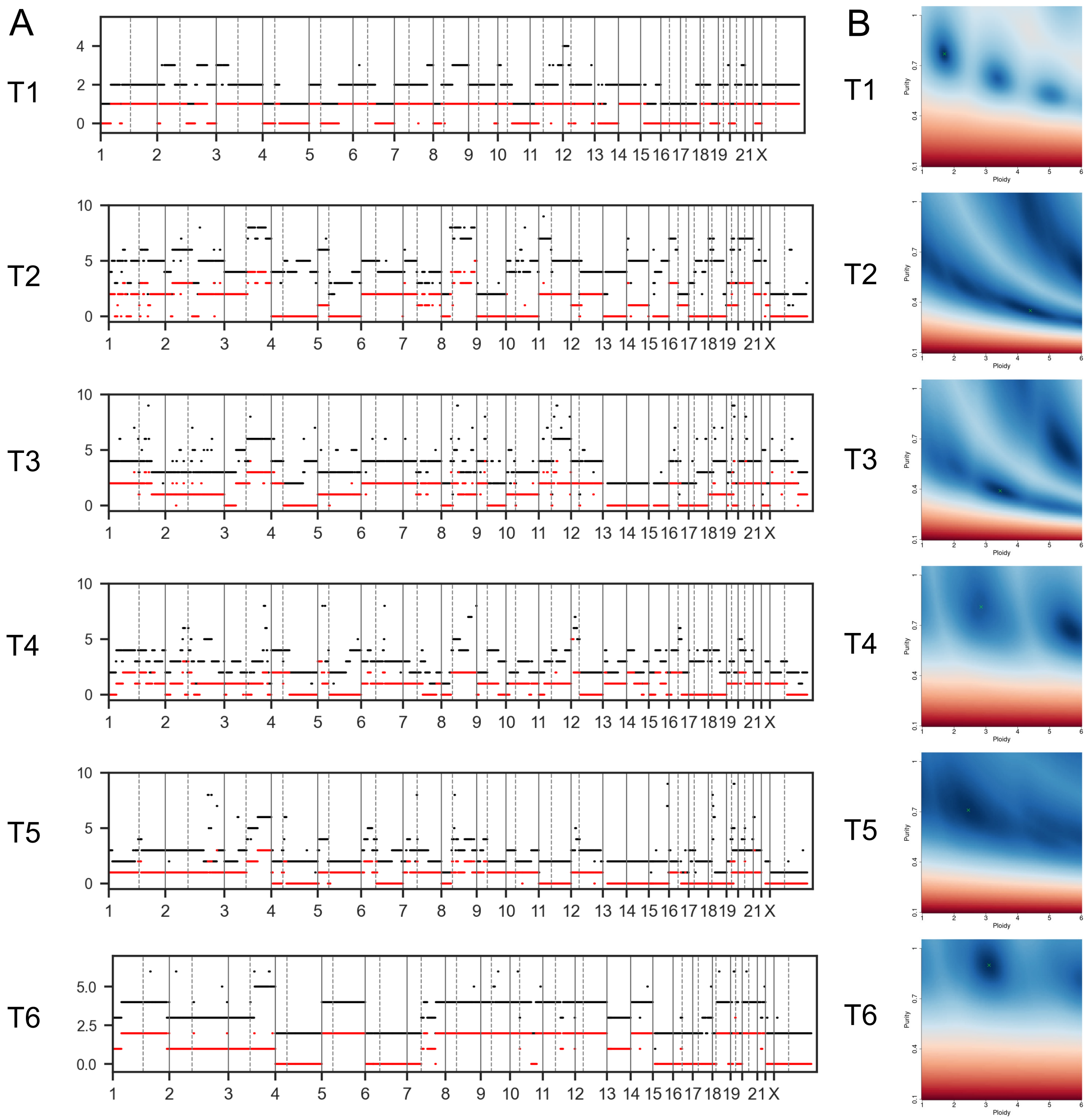

Purity	Ploidy
77	1.80
35	4.57
39	3.56
81	3.09
71	2.61
90	3.26

A) Allele-specific copy number variations in tumor samples (T1-T6).
The x-axis represents genomic positions across chromosomes 1–22 and X, with vertical dotted lines indicating centromere positions. The y-axis shows copy number values. Red and black lines represent the estimated minor and total allele copy numbers, respectively.

B) Sunrise plots for estimating tumor purity and ploidy using the default parameter settings.
The corresponding estimated purity (%) and ploidy values for each tumor are shown in the adjacent table.

Supplementary Figure 4: Overlaps of detected structural variants between four somatic callers

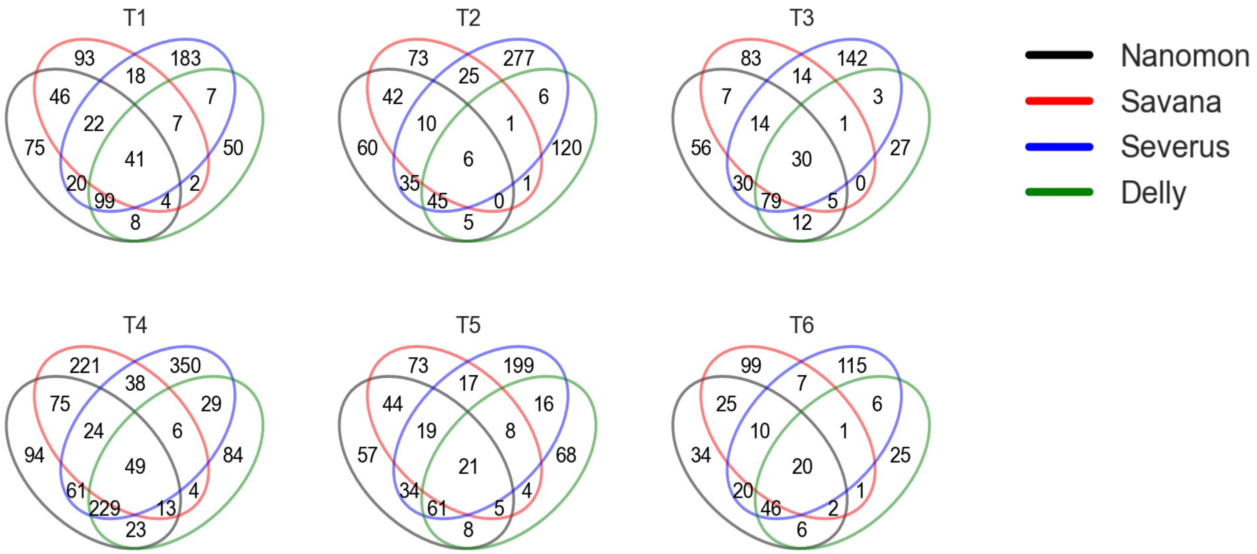

Venn diagrams display the number of structural variants (SVs) identified by each of the four SV detection tools—nanomonsv [4] (black), SAVANA [5] (red), Severus [6] (blue), and DELLY [7] (green)—in tumor samples T1 through T6. Simple SVs (e.g., deletions, duplications, inversions, translocations) were standardized using AnnotSV [8] to enable consistent comparison across tools. While a subset of SVs was commonly detected by multiple callers (shown in the overlapping regions), a considerable number of SVs were uniquely detected by individual tools.
These differences likely reflect the distinct algorithmic frameworks and variant calling strategies by each tool, which underscores the potential benefit of integrative or consensus-based approaches for comprehensive SV detection in long-read somatic variant analysis.

Supplementary Figure 5: Complex structural variants detected using JaBbA [9]

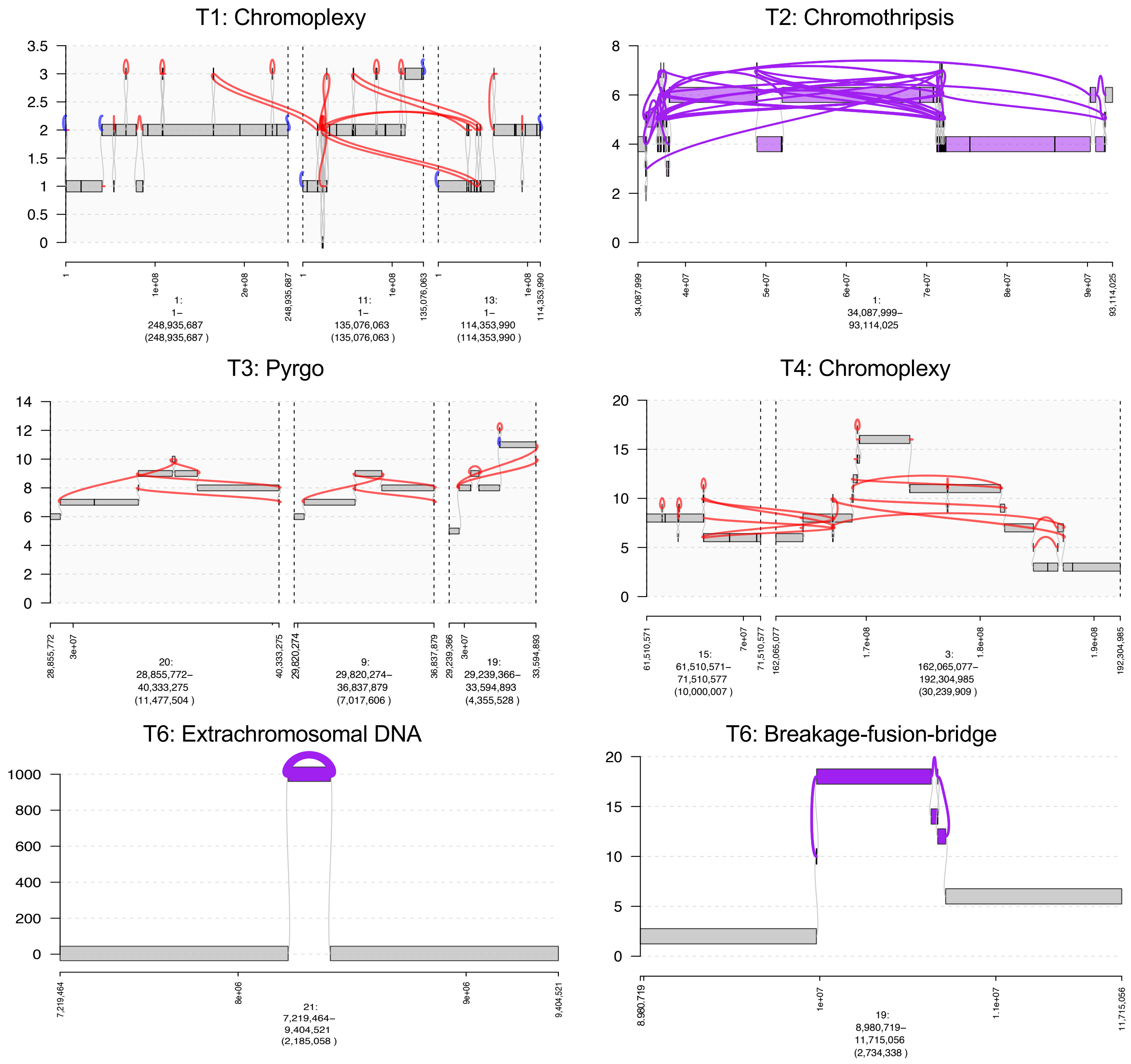

Representative examples of complex structural variants detected in tumor samples T1–T6 using JaBbA [9]. Each panel illustrates distinct types of complex rearrangements. T1 and T4: Chromoplexy, characterized by chained inter- and intra-chromosomal rearrangements involving multiple breakpoints and copy number alterations. T2: Chromothripsis, defined by clustered rearrangements and oscillating copy number states across a limited genomic region. T3: Pyrgo, indicating localized rearrangements resulting in focal gains. T6 (left): Extrachromosomal DNA, identified as highly amplified, circular segments not integrated into chromosomes. T6 (right): Breakage–fusion–bridge cycle, featuring stepwise copy number increases and rearrangements consistent with iterative cycles of telomere erosion and fusion. Red arcs represent rearrangements with positive junction copy numbers; purple arcs denote complex inversional or duplicative junctions. Grey bars indicate genomic segments, with vertical axis showing segmental copy numbers.

Supplementary Figure 6: Read coverage of centromeric regions

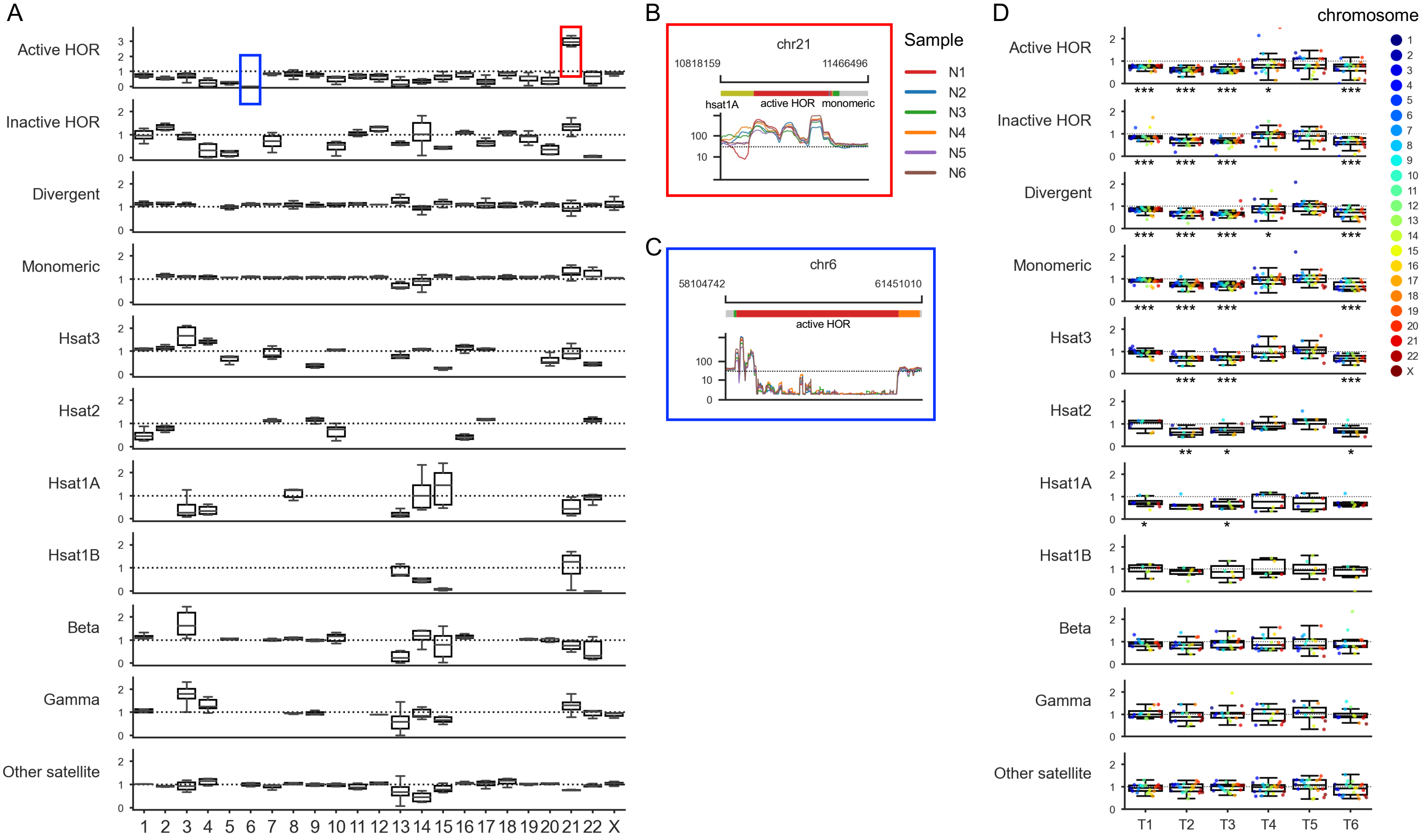

A) Normalized median read coverage of 1kb windows for each centromeric satellite type across chromosomes in six normal samples.
The black dotted line indicates the median genome-wide read coverage for each sample. Active HORs on chromosome 21 (red box) and 6 (blue box) are shown in detail in panel B and C, respectively.

B) Detailed read coverage in the active HOR region of chromosome 21, which showed high coverage.
The Y-axis represents the average read coverage in 10K bp window for each sample

C) Detailed read coverage in the active HOR region on chromosome 6, which showed low coverage.
The Y-axis represents the average read coverage in 10K bp window for each sample

D) Tumor-to-normal read coverage ratios for each centromeric satellite type per chromosome.
Ratios were calculated as the proportion of reads in each region relative to the whole genome, and compared between tumor and matched normal samples using chromosome-paired t-tests. Asterisks indicate P-values: <0.05 (*), <0.01 (**), < 0.001 (***). The black dotted line indicates a coverage ratio of 1.0, equivalent to the corresponding normal sample.

Supplementary Figure 7: Validation of methylation profiles of the studied samples

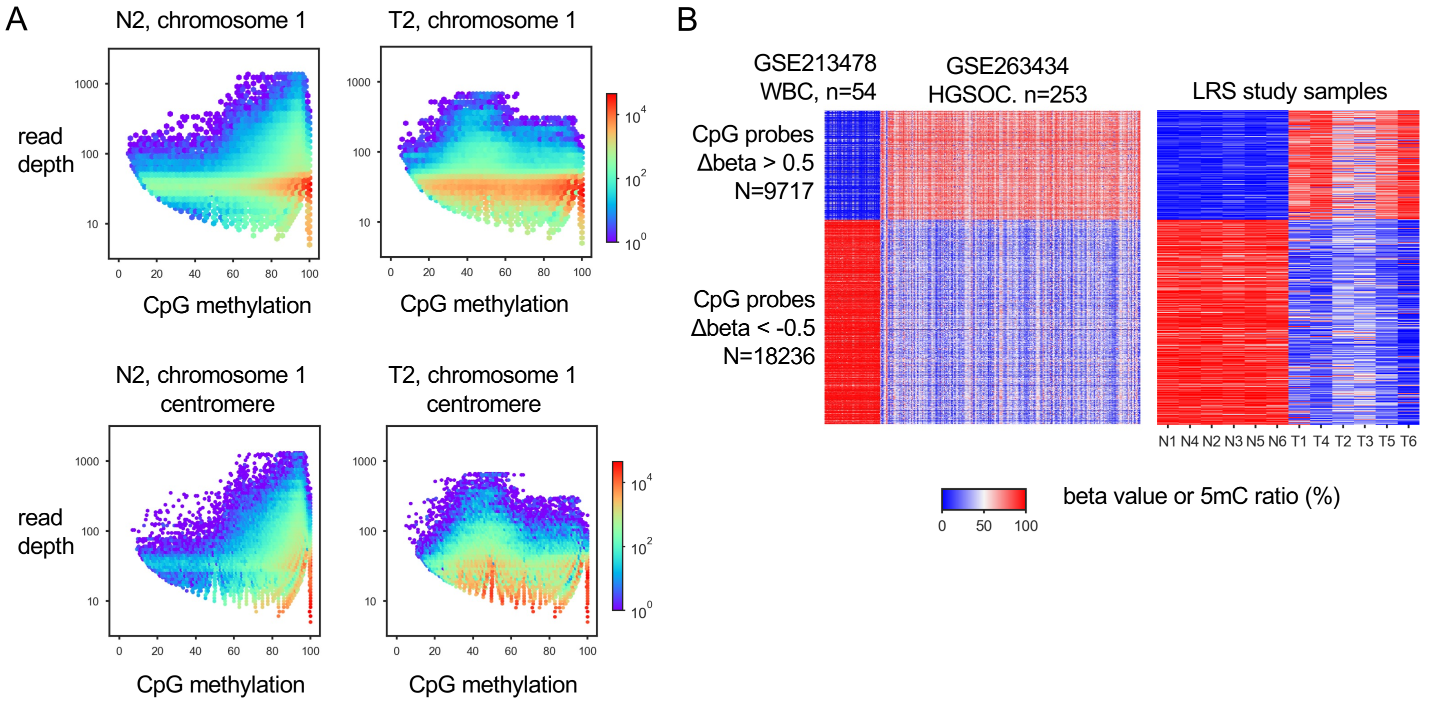

A) No significant correlation was observed between CpG methylation and read depth.
An example in whole chromosome 2 (upper) and centromeric regions (lower) of N2 (left) and T2 (right).
Color intensity indicates the number of data points per bin.
No significant correlation was found using either Spearman's rank correlation or Pearson's correlation coefficient.

B) Methylation of CpG sites differentially methylated between HGSOCs and whole blood cells using external data.
After extracting differentially methylated probes and converting the genomic loci for LRS data (see Supplementary Methods), similar methylation patterns were observed in our LRS samples.

Supplementary Figure 8: Detailed CpG methylation analysis for each chromosome centromere

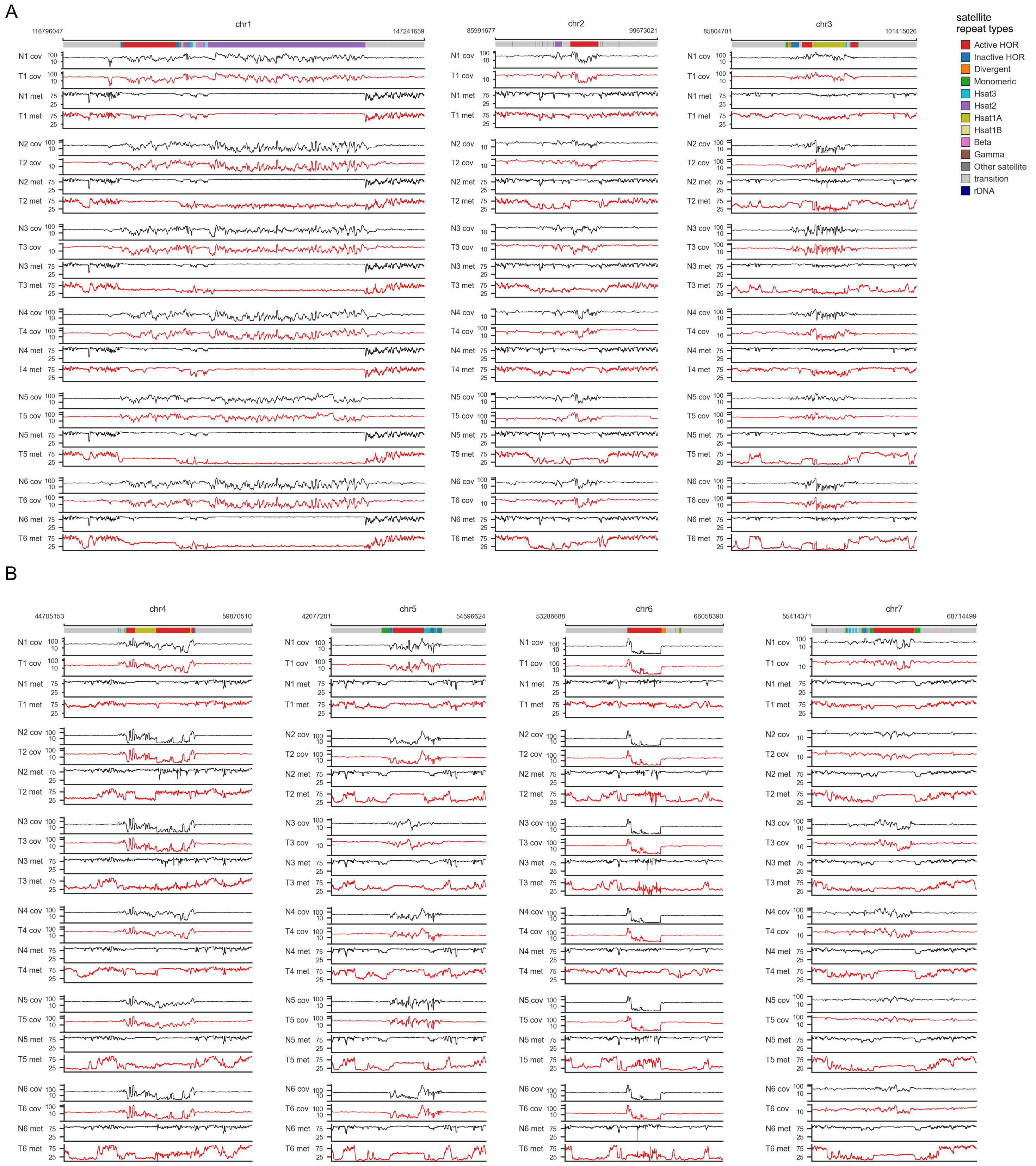

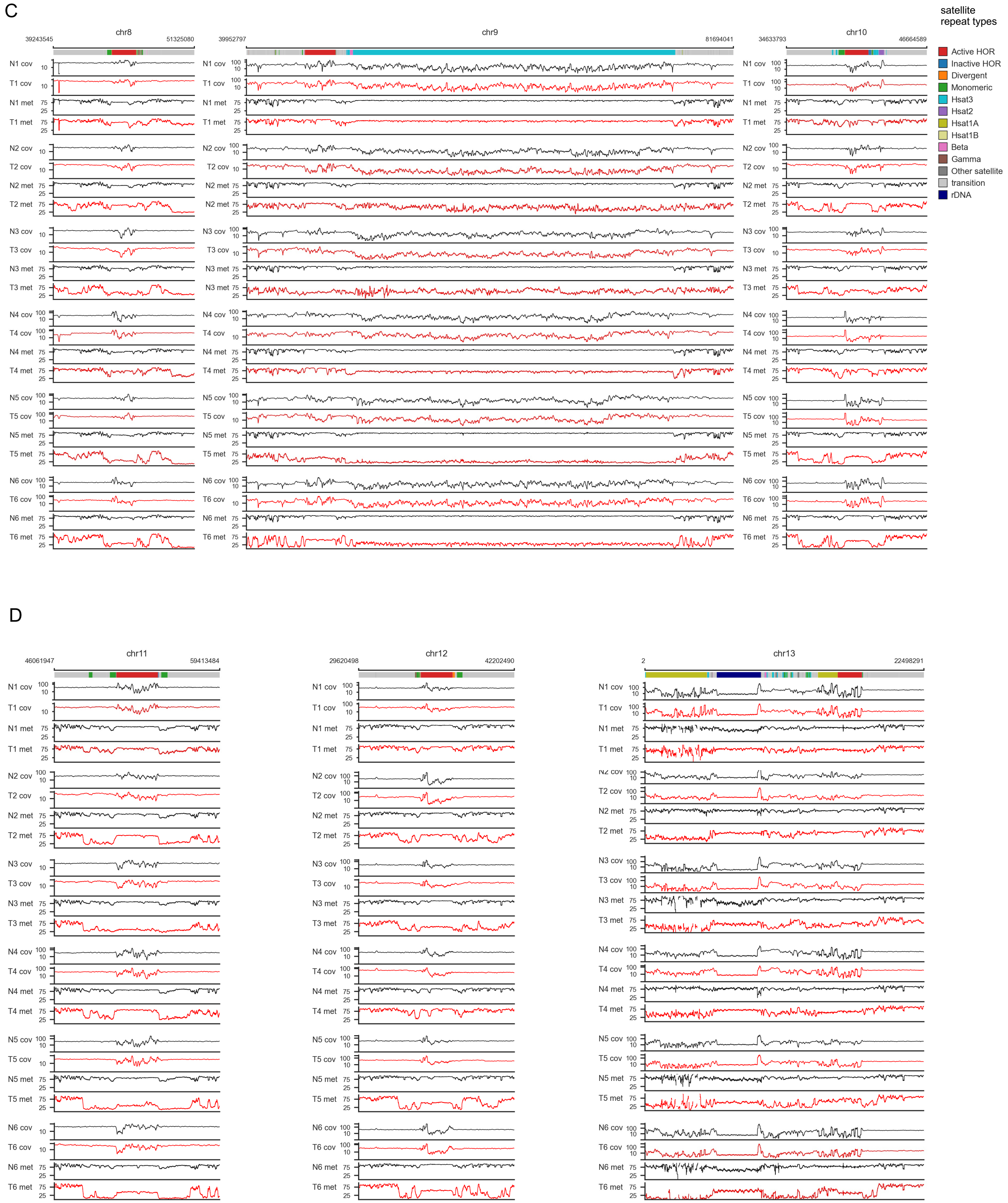
C
chr8
39243545
51325080
chr9
39952797
81694041
chr10
34633793
46664589
satellite repeat types
Active HOR
Inactive HOR
Divergent
Monomeric
Hsat3
Hsat2
Hsat1A
Hsat1B
Beta
Gamma
Other satellite
transition
rDNA
N1 cov
T1 cov
N1 met
T1 met
N2 cov
T2 cov
N2 met
T2 met
N3 cov
T3 cov
N3 met
T3 met
N4 cov
T4 cov
N4 met
T4 met
N5 cov
T5 cov
N5 met
T5 met
N6 cov
T6 cov
N6 met
T6 met
D
chr11
46061947
59413484
chr12
29620498
42202490
chr13
2
22498291

E

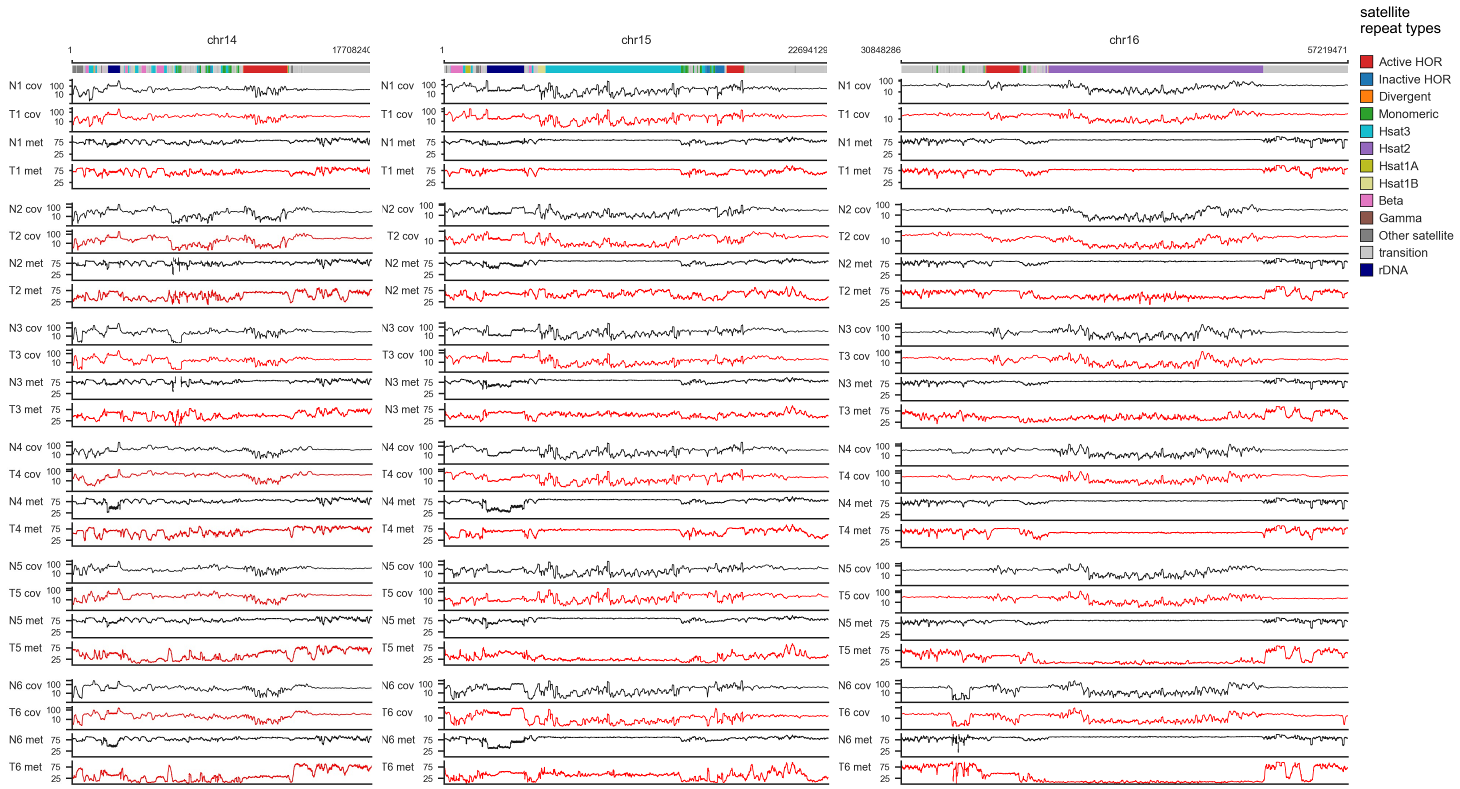
chr14
1
17708240
chr15
1
22694129
chr16
30848286
57219471
N1 cov
T1 cov
N1 met
T1 met
N2 cov
T2 cov
N2 met
T2 met
N3 cov
T3 cov
N3 met
T3 met
N4 cov
T4 cov
N4 met
T4 met
N5 cov
T5 cov
N5 met
T5 met
N6 cov
T6 cov
N6 met
T6 met
satellite repeat types
Active HOR
Inactive HOR
Divergent
Monomeric
Hsat3
Hsat2
Hsat1A
Hsat1B
Beta
Gamma
Other satellite
transition
rDNA

F

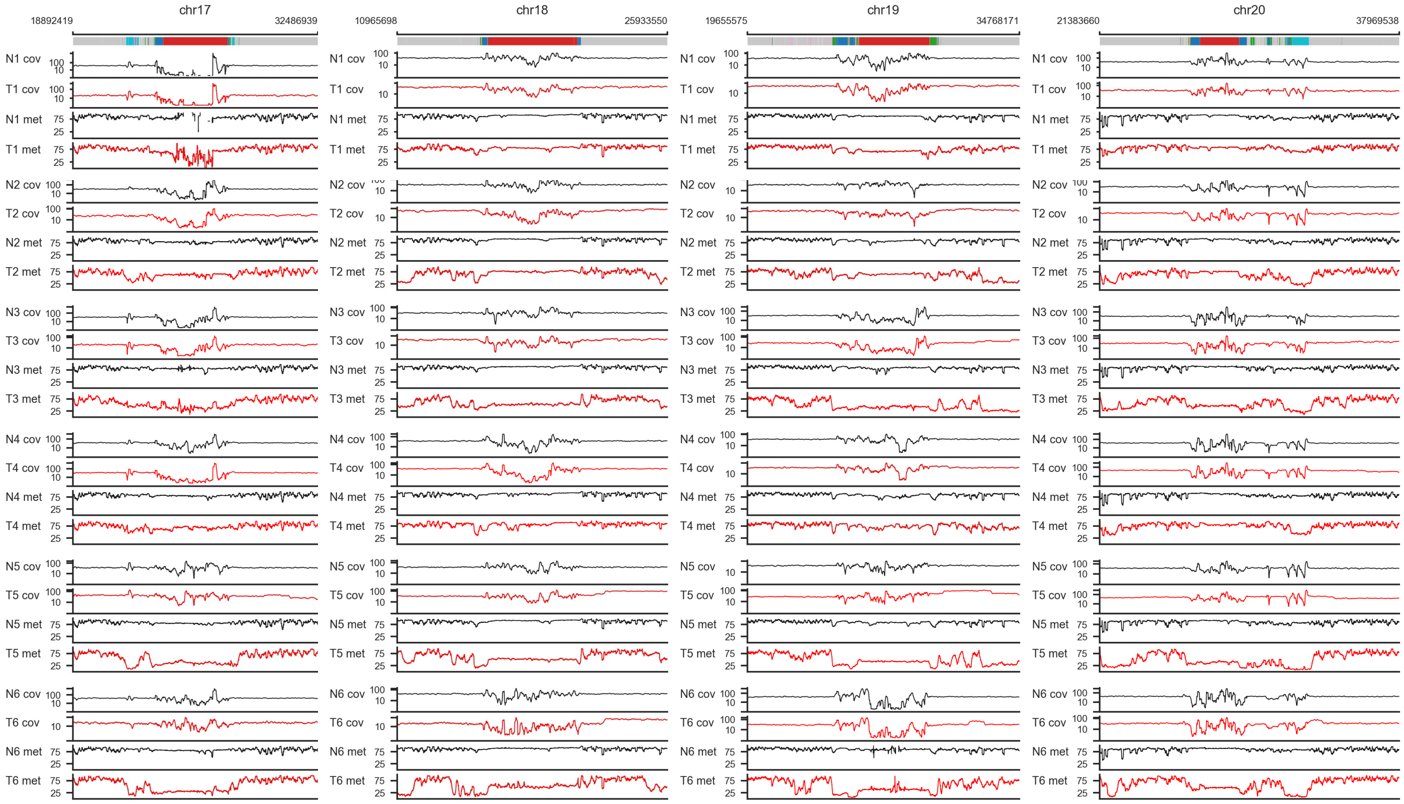
chr17
18892419
32486939
chr18
10965698
25933550
chr19
19655575
34768171
chr20
21383660
37969538
N1 cov
T1 cov
N1 met
T1 met
N2 cov
T2 cov
N2 met
T2 met
N3 cov
T3 cov
N3 met
T3 met
N4 cov
T4 cov
N4 met
T4 met
N5 cov
T5 cov
N5 met
T5 met
N6 cov
T6 cov
N6 met
T6 met

G

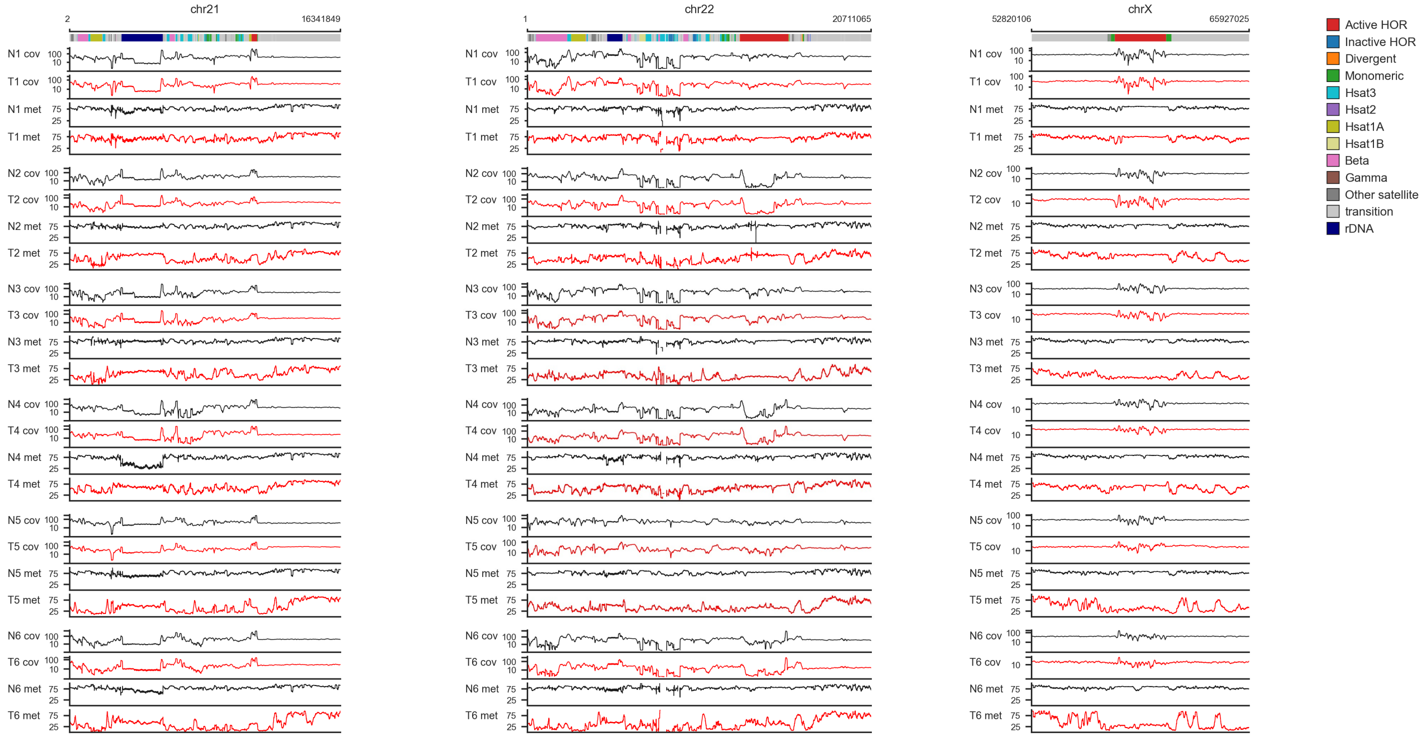

Panels A–G show chromosomes 1–3 (A), 4–7 (B), 8–10 (C), 11–13 (D), 14–16 (E), 17–20 (F), and 21, 22, X (G). At the top of each panel, the centromeric coordinates based on the T2T-CHM13 reference genome are shown along with the corresponding satellite repeat types. For each chromosome, the plots display CpG site coverage (cov) and methylation levels (met) in normal (N) and tumor (T) samples, calculated as a 10-kb sliding window average.

Supplementary Figure 9: Estimated telomere length per chromosome arm calculated by Telometer [10]

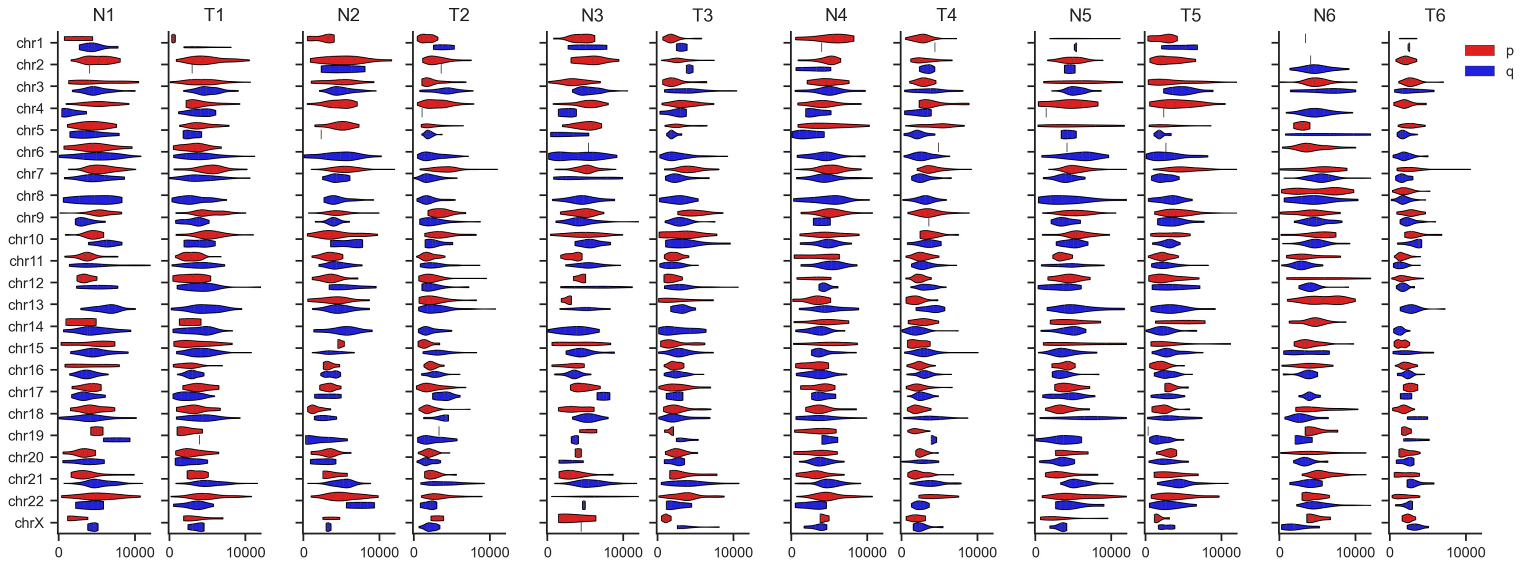

Estimated telomere length per chromosome arm.
Violin plots represent the estimated telomere length for each chromosome arm in normal (N1–N6) and tumor (T1–T6) samples,with red and blue representing the short (p) and long (q) arms, respectively.

Supplementary Figure 10: Telomere length shortening in tumor samples analyzed by Telogator2 [11]

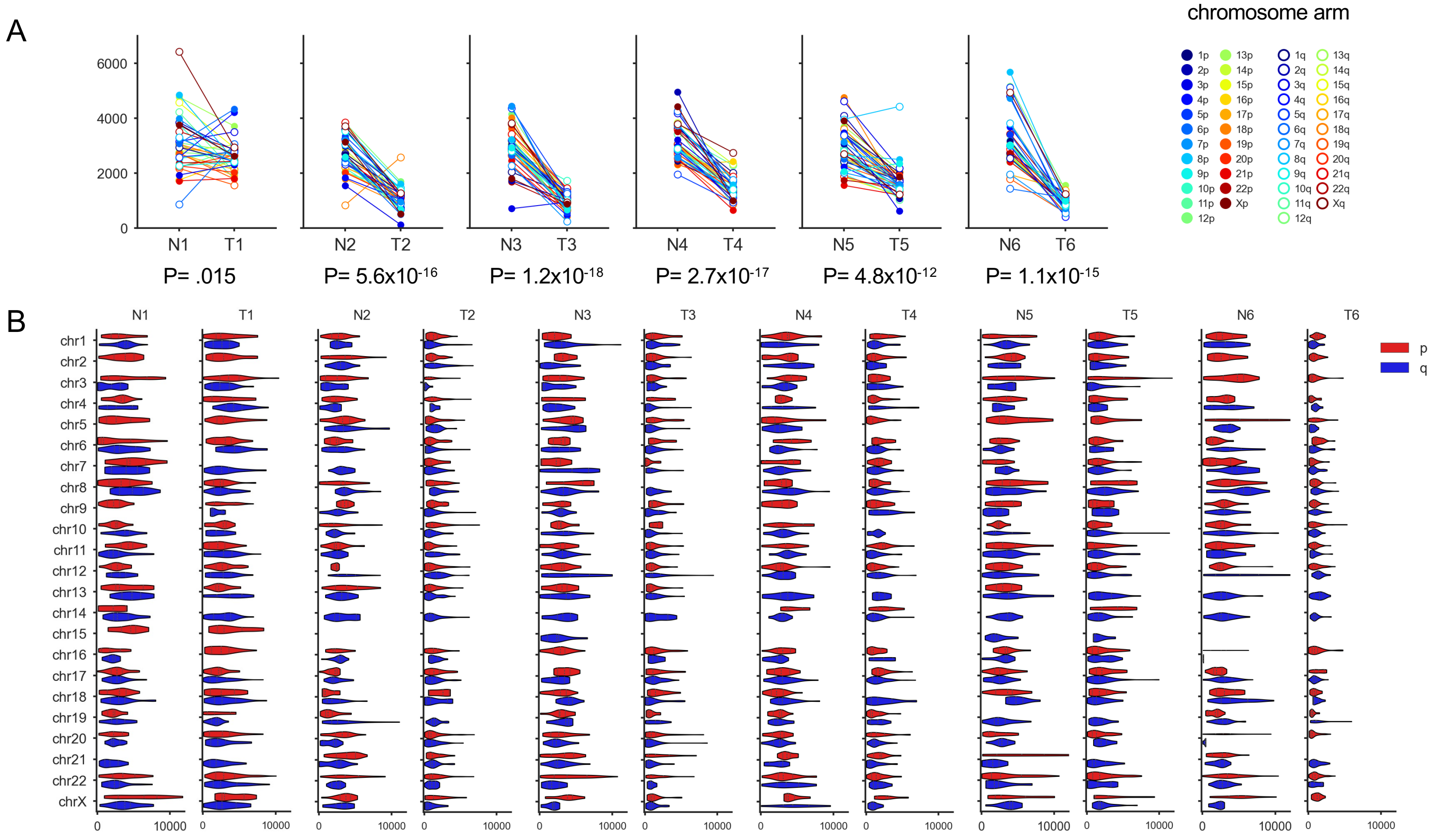

A) Estimated telomere length per chromosomal arm in matched normal (N) and tumor (T) samples. Each point represents a specific chromosome arm, with lines connecting corresponding arms in paired samples. The estimated telomere length was significantly shorter in tumor samples than in matched normal samples in all cases. P-values were calculated by paired t-test.

B) Estimated telomere length per chromosome arm. Violin plots represent the estimated telomere length for each chromosome arm in normal (N1–N6) and tumor (T1–T6) samples,with red and blue representing the short (p) and long (q) arms, respectively.Red and blue represent the short and long arms of each chromosome, respectively.

Supplementary Figure 11: Allele-specific and non-specific THOR hypermethylation

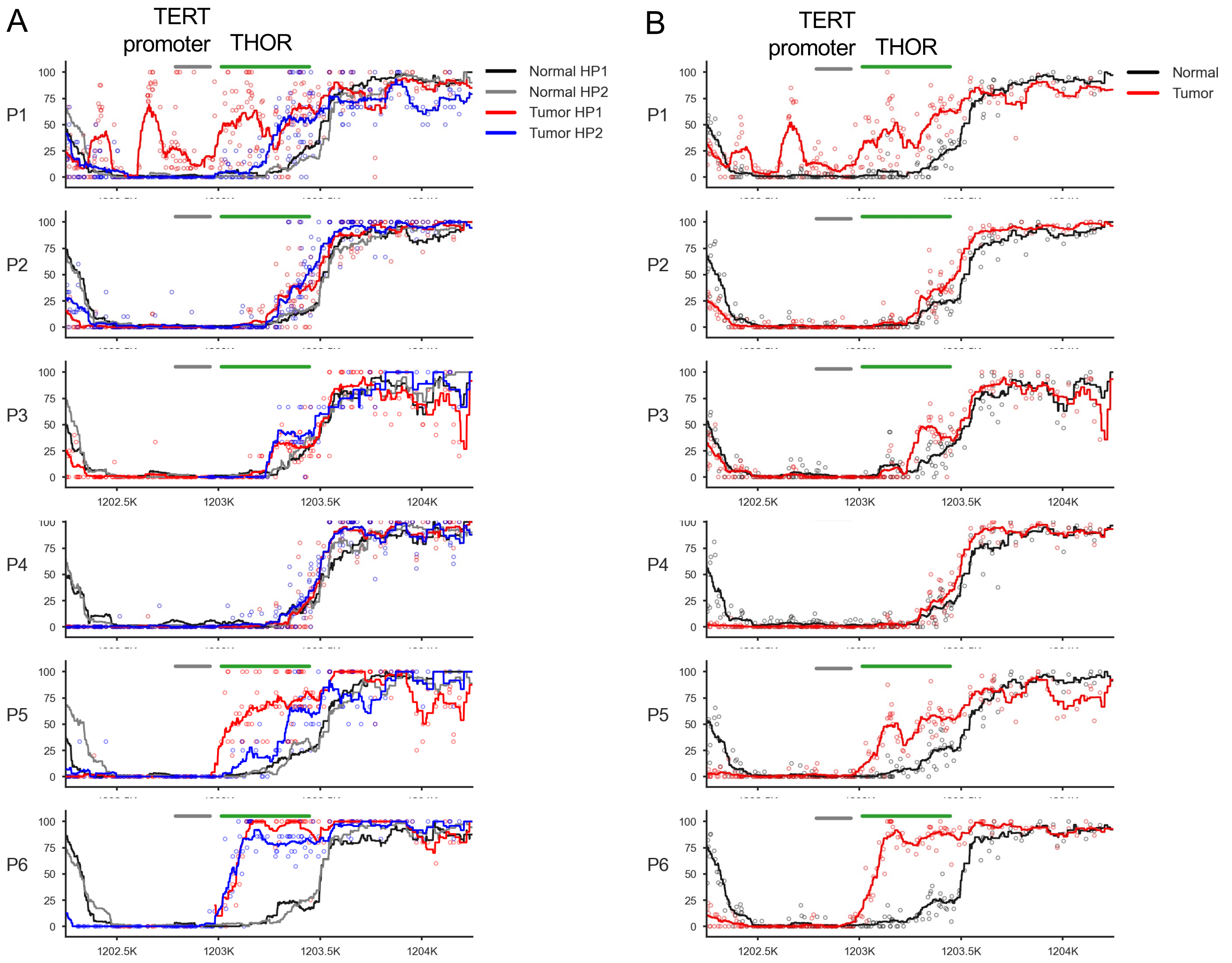

A) Allele-specific CpG methylation analysis of the TERT hypermethylated oncological region (THOR) in six tumor–normal pairs (P1–P6).
T6 exhibited biallelic THOR hypermethylation, whereas T1 and T5 showed monoallelic hypermethylation. Dots represent methylation levels at individual CpG sites (black: normal haplotype 1 [HP1], grey: normal haplotype 2 [HP2], red: tumor HP1, blue: tumor HP2). Solid lines show smoothed methylation profiles (100-bp sliding window). CpG sites with <3× coverage were excluded.

B) Non-allele-specific methylation analysis of the same regions. Monoallelic hypermethylation in T1 and T5 is difficult to detect without haplotype resolution. Dots represent methylation levels at individual CpG sites (black: normal, red: tumor). Solid lines show smoothed methylation profiles (100-bp sliding window). CpG sites with <5× coverage were excluded.

Supplementary Tables

Supplementary Table 1: Patient Characteristics

Case ID	Age	FIGO Stage	Surgery	Residual	Clinical HRD test	Recurrence	PFS	Death	OS
P1	51	IIIA2	PDS	R0	Germline BRCA1, c.2138C>G	1	2174	0	2447
P2	53	IIIC	PDS	R0	-	1	923	0 (sensor)	1299
P3	73	IIIB	PDS	R1	-	1	1511	1	2065
P4	44	IIIB	PDS	R0	Germline BRCA1, c.4524G>A	1	1303	0	2321
P5	65	IIIA	PDS	R0	-	1	186	1	435
P6	68	IIIC	PDS	R0	-	1	508	1	1531

Demographic and clinical information for six patients, including age, FIGO stage, surgery type, residual tumor status, HRD test results, recurrence, progression-free survival (PFS), and overall survival (OS)

Supplementary Table 2: Quality Control Metrics for Oxford Nanopore Long-Read Sequencing

Case ID	Specimen	Sample ID	Raw		Q score >10		N50 (kb)	Median coverages
			Bases (Gb)	Reads (M)	Bases called (Gb)	Reads called(M)		
P1	White blood cell	N1	139.68	22.58	120.75	16.83	8.591	37.96
	Ovarian tumor	T1	119.84	27.89	102.92	20.12	7.807	32.64
P2	White blood cell	N2	111.14	16.64	106.11	14.5	8.876	33.06
	Ovarian tumor	T2	105.67	30.14	91.87	18.51	7.003	28.48
P3	White blood cell	N3	111.81	18.17	104.66	15.15	8.223	32.64
	Ovarian tumor	T3	105.76	33.42	89.13	19.36	6.394	27.64
P4	White blood cell	N4	132.53	26.14	114.92	18.12	8.239	36.30
	Ovarian tumor	T4	168.87	61.92	138.88	33.55	5.954	34.17
P5	White blood cell	N5	131.97	19.17	115.81	16.87	9.141	36.11
	Ovarian tumor	T5	121.74	26.79	110.31	24.94	6.14	34.23
P6	White blood cell	N6	144.6	18.57	127.48	16.71	9.772	39.77
	Ovarian tumor	T6	92.4	19.82	82.21	17.99	6.505	25.48

Supplementary Methods

Analysis pipeline overview

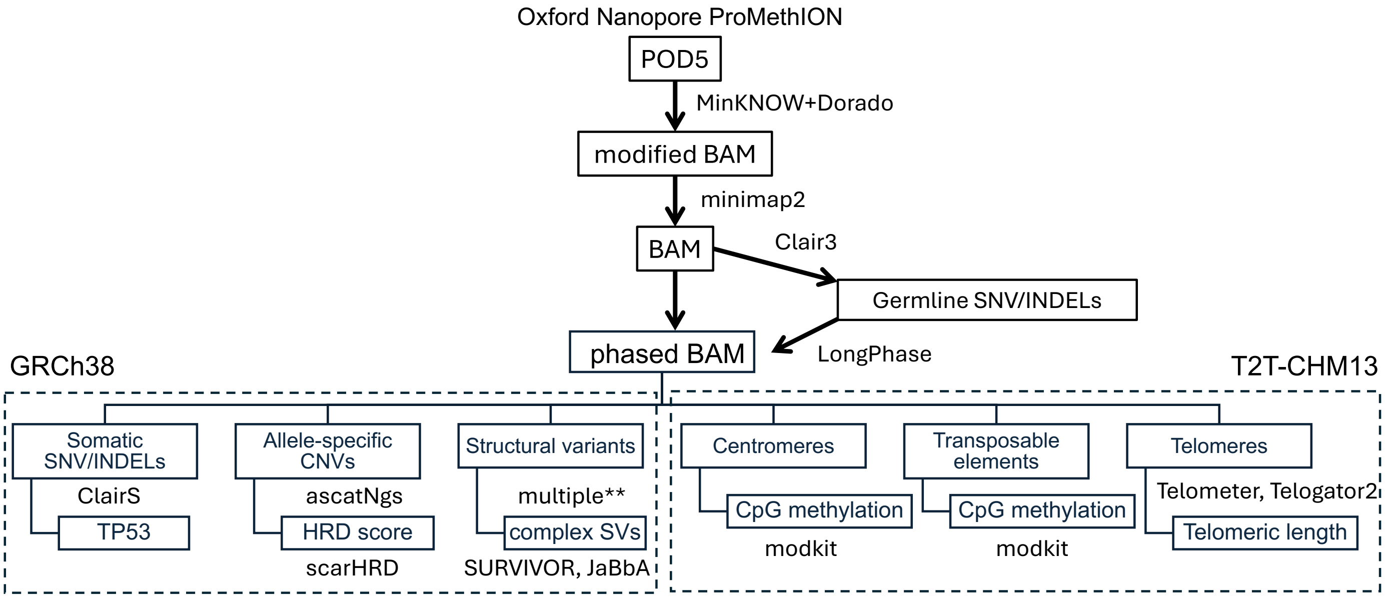

** Severus, SAVANA, nanomonsv and DELLY

Sample collection, preparation, and long-read whole genome sequencing

HGSOC tumors with sufficient tumor purity, as determined by a pathologist (B.L.) based on the diagnostic hematoxylin and eosin specimen, were selected for analysis. Genomic DNA was extracted from the cryopreserved pre-treatment tumor tissues and matched normal blood samples stored in the institutional tissue bank at MD Anderson Cancer Center. 3 ug of Qubit quantified genomic DNA was sheared to 5-10 kilobase (kb) using Covaris g-tubes. Following cleanup with Ampure beads, 1.5 ug of the fragmented DNA was used to prepare libraries using the SQK-LSK114 kit per the manufacturers protocol. Briefly, the DNA was repaired using the NEBNext FFPE DNA Repair mix and end-repaired using the Ultra II End-Prep Enzyme Mix. Following Ampure bead clean-up, adapters were ligated. The resultant libraries were quantified using the Qubit HS assay and the concentrations adjusted to load thirty to fifty fmol of each final library to a ONT R10.4.1 flow cell. The flow cells were run on the PromethION24 using Super accurate basecallling with methylation, for up to 72 hours with 2 washes and two additional loadings. POD5 files were generated by MinKNOW [12] software and then processed into modified BAM files by Dorado basecaller [13].

Detection of pathogenic SNVs/INDELs

Sequencing data from tumor and normal tissues were mapped to the GRCh38 reference genome using minimap2 [14]. Germline SNV/INDELs were identified using Clair3 [15] with LongPhase [16], while somatic SNV/INDELs were detected using ClairS [1] with the 1041_e82_400bps_sup_v430 model. Variants passing default quality filters were annotated using Ensembl-VEP [17]. Variants classified as “pathogenic” or “likely pathogenic” in ClinVar and “Oncogenic” or “Likely Oncogenic” in OncoKB [18] (accessed on November 15, 2024) were retained. Mutations with allele frequencies <1% in all populations were excluded. Truncating mutations (variant types of ‘nonsense’, ‘nonstop’, and ‘frameshift INDELs’) were retained. The functional impact of missense mutations was assessed using SIFT [19] and PolyPhen [20], and those likely to be damaging or pathogenic were retained. Mutations in splicing sites and transcription start sites were retained when annotated as pathogenic in spliceAI [21]. All detected single base substitutions (SBSs) were grouped into 96 patterns based on flanking bases using SigProfilerExtractor [22], and then decomposed into components with the COSMIC SBS signatures v3.4 [23] as a reference by non-negative least squares (NNLS). The ratio of

each signature to the total number of SBSs was calculated for each sample.

For Clair3, we used apptainer,
```
sif=path/to/clair3_109.sif
command="run_clair3.sh"
in1=path/to/inputBAM
in2=path/to/hg38.fa
in3=path/to/rerio/clair3_models/r1041_e82_400bps_sup_v430
in4=path/to/inputBAMindex
in5=path/to/hg38.fa.fai
out=`pwd`
apptainer exec \
  -B $in1,$in2,$in3,$in4,$in5 \
  -B $out:/output \
  $sif $command \
  --bam_fn=$in1 \
  --ref_fn=$in2 \
  --platform="ont" \
  --model_path=$in3 \
  --threads=12 \
  --output=/output/$2 \
  --enable_phasing \
  --longphase_for_phasing \
  --use_longphase_for_intermediate_phasing \
  --use_longphase_for_final_output_phasing
```

For ClairS,
```
ClairS/run_clairs \
  -T path/to/tumor.bam \
  -N path/to/normal.bam \
  -R path/to/hg38.fa \
  -t 12 \
  -p ont_r10_dorado_sup_4khz \
  -o ${OUTPUT_DIR} \
  --enable_indel_calling
```

Allele-specific copy number variation, tumor purity, ploidy and the HRD score

Using sequencing data mapped to GRCh38, ascatNgs [3] was applied in tumor-normal paired mode with default GC content and replication timing corrections. For long-read sequencing data, alleleCounter [24] was applied with the following recommended options [25]: loci_binsize = 500, min_base_qual= 10, additional_allelecounter_flags="-f 0". The HRD score was calculated using scarHRD [26], summing the TAI, LST, and LOH scores to obtain the HRD-sum.
```
## Rscript
library(ASCAT)
ac='path/to/alleleCounter'
args=commandArgs(trailingOnly=T)
locis='path/to/G1000_loci_WGS_hg38_chr/G1000_loci_hg38_chr'
alleles='path/to/G1000_alleles_hg38_chr'
ascat.prepareHTS(
  tumourseqfile = ‘path/to/tumorBAM’,
  normalseqfile = ‘path/to/normalBAM’,
```

```
  tumourname = ‘tumor_name’,
  normalname = ‘normal_name’,
  allelecounter_exe = ac,
  skip_allele_counting_normal = FALSE,
  skip_allele_counting_tumour = FALSE,
  alleles.prefix = locis,
  loci.prefix = alleles,
  gender = gender,
  genomeVersion = "hg38",
  nthreads = 12,
 tumourLogR_file = ‘path/to/sample_tumor_LogR.txt’,
 tumourBAF_file = ‘path/to/sample_tumor_BAF.txt’,
 normalLogR_file = ‘path/to/sample_normal_LogR.txt’,
 normalBAF_file = ‘path/to/sample_normal_BAF.txt’,
  loci_binsize = 500,
  min_base_qual= 10,
  additional_allelecounter_flags="-f 0")
```

Detection of somatic structural variants (SVs)

A previous study reported that the detection of somatic SVs in cancer using long-read sequencing showed better performance when employing somatic SV-specific callers than when using a general SV detection approach based on tumor-normal subtraction [27]. And several studies recommended integrateing multiple tools show better performance for somatic SV detection [28,29]. Accordingly, we used four different somatic SV callers, including Severus [6], SAVANA [5], nanomonsv [4], and DELLY [7] with default parameter settings. Variants smaller than 50 bp were excluded.

For Nanomonsv,

```
panel=path/to/hprc_year1_data_freeze_nanopore_minimap2_2_24_merge_control
nanomonsv parse $normal_bam ${sample}_Normal
nanomonsv parse $tumor_bam ${sample}_Tumor
nanomonsv get ${sample}_Tumor $tumor_bam --control_prefix ${sample}_Normal --control_bam
$normal_bam --control_panel_prefix $panel path/to/hg38.fa --use_racon --process 12 --
single_bnd
```

For Severus,

```
vntr=path/to/human_GRCh38_no_alt_analysis_set.trf.bed ## [30]
severus \
 --target-bam $tumor_bam \
 --control-bam $normal_bam \
 -t 24 \
 --out-dir ${sample} \
 --vntr-bed $vntr
```

For SAVANA,

```
contigs=path/to/contigs.chr.hg38.txt
savana --tumour $tumor_bam --normal $normal_bam --outdir $sample --ref path/to/hg38.fa --ont
--contigs $contigs --no_blacklist --single_bnd --max_ploidy 5.5
```

For DELLY,

```
exc=path/to/delly/excludeTemplates/human.hg38.excl.tsv
delly=path/to/delly_v1.3.1_linux_x86_64bit
## call tumor + normal
$delly lr -y ont -g path/to/hg38.fa-o ${sample}_sv.bcf -x $exc $tumor_bam $normal_bam
## make sample table
nname=`basename $normal_bam |sed -e s/\.bam//g`
```

```
tname=`basename $tumor_bam |sed -e s/\.bam//g`
echo -e "$tname\ttumor\n$nname\tcontrol\n" > ${sample}_table.tsv
## filter for somatic SVs
$delly filter -p -f somatic -o ${sample}_somatic.bcf -s ${3}_table.tsv ${sample}_sv.bcf
# extract PASS variants
bcftools view -f PASS ${3}_somatic.bcf > ${3}_somatic.vcf
```

Output VCF files were converted to the same format using AnnotSV [8], and SVs detected by at least two callers of the four were retained as simple SVs using SURVIVOR [31] merge function.

For AnnotSV,

```
ANNOTSV=path/to/AnnotSV_dir
$ANNOTSV/bin/AnnotSV -SVinputFile $inVCF -SVinputInfo 1 -outputDir $sample -outputFile
$prefix -vcf 1 -annotationMode full
```

For SURVIVOR,

```
SURVIVOR merge path_to_vcf_files 1000 1 1 1 0 50 sample_merged.vcf
```

Simple SVs were grouped into 32 patterns using SigProfilerExtractor [22] and decomposed into components of COSMIC V3.4 SV signatures [23] by NNLS. Complex SVs were identified using JaBbA [9] with the read coverage generated using CNVkit [32] and all detected SVs from the four callers as input.

For CNVkit

```
cnvkit.py batch $tumor_bam -n $normal_bam -m wgs --output-dir $outDir \
 -p 12 --fasta path/to/hg38.fa --target-avg-size 10000
```

For JaBbA, we used Gurobi 11.0.0 [33]

```
jba $inputVCF $inCov.txt -n $prefix --cores 12 --gurobi TRUE
```

Centromeres and Transposable elements (TEs)

Sequencing reads were mapped to the T2T-CHM13 reference genome using minimap2 [14], and genomic annotations for centromeres and satellite repeats were obtained from a T2T study [34]. Base-level read coverage was calculated using samtools [35] and mosdepth [36]. The median read coverage for centromeric regions was computed per 1-kb genomic bin and normalized to the genome-wide median coverage for each sample.

Repeat sequences annotations of major TEs, including Long Interspersed Nuclear Element-1 (LINE1) Human-Specific subfamily (L1HS), Alu short interspersed nuclear element (SINE) subfamily Y (AluY), SINE-VNTR-Alu (SVA), and Endogenous RetroVirus (ERV) were obtained from a T2T study [37]. To analyze full-length of these insertions, we selected those with lengths >6 kb for L1HS (n=355), >300 bp for AluY (n=58288), >1 kb for SVA (n=2435), >1kb for ERV (n=271). The median read coverage was calculated per 1-kb for L1HS, SVA and ERV, and per insertion for AluY, normalized to the genome-wide median coverage for each sample.

Quantification of 5-methylcytosine (5mC) levels in CpG sites, centromeres, and TEs

Sequence data mapped to the T2T-CHM13 reference genome with base modification information were processed using modkit [45] to quantify 5mC levels across all genomic CpG sites.

```
modkit pileup $inBAM $sample_pileup.bed --ref path/to/hs1.fa --cpg --ignore h
```

Methylation data for specific regions, including centromeric regions, inserted TE regions, and the gene loci of BRCA1, RAD51C, and TERT, were extracted using bedtools [46].

```
target_bed=path/to/target.bed
bedtools intersect -a $sample_pileup.bed -b $target_bed > target_pileup.bed
```

For centromeres and TE regions, CpG methylation levels were assessed as the average 5mC ratio (methylated reads / total reads) within each 1-kb genomic bin, while for AluY elements, the ratio was computed per insertion.

Haplotype-specific methylation analysis of the *TERT* promoter and *TERT* hypermethylated oncological region (THOR)

Haplotype phasing was performed using Clair3 [15] and LongPhase [16] (see also **Detection of pathogenic SNVs/INDELs**).

```
longphase=path/to/longphase_linux-x64_v1.7.3
$longphase haplotag -s $SampleVCF -r path/to/hs1.fa -b $inBAM -t 12 -o $phasedBAM
```

After generating phased BAM files, haplotype-specific CpG methylation were computed using modkit [38].

```
modkit pileup $phasedBam $outDir --ref path/to/hs1.fa -t 12 \
--cpg --ignore h --partition-tag HP --prefix $prefix
```

CpG methylation information was extracted for the regions of the TERT promoter and THOR: chr12:1,200,000-1,204500 using bedtools [39].

```
echo -e "chr12\t1202000\t1204500" > region.bed
bedtools intersect -a $sample_pileup.bed -b region.bed -wo > sample_tert.bed
```

For each 100-bp bin, CpG sites with fewer than 3 reads covering all CpG sites within the bin were excluded from the analysis.

Telomere length

Telomere length for each chromosome arm was estimated using Telometer [10] and Telogator2 [11], based on reads mapped to the T2T-CHM13 genome supplemented with subtelomeric sequences obtained from a previous study [40]

First, we remapped sequencing reads

```
## make fasta
cat hs1.fa stong_subtels.fa > t2t-and-subtel.fa
samtools faidx t2t-and-subtel.fa
## make minimap2 index
minimap2 -t 12 -d t2t_subtel.mmi t2t-and-subtel.fa
## re-mapping
ref_fa=path/to/t2t-and-subtel.fa
samtools fastq -T MM,ML $inBAM -@ 24 | minimap2 -ax map-ont -t 24 -y $ref_fa - | samtools
view -@ 24 -bS -o ${sample}.bam
samtools sort -@ 24 ${sample}.bam -o ${sample}_sorted.bam
samtools index -@ 24 ${sample}_sorted.bam
```

For Telometer,

```
ref_fa=path/to/t2t-and-subtel.fa
telometer -b ${sample}_sorted.bam -o ${output}.tsv -m 4000 -g 100 -t 24 --memlimit 64
```

For Telogator2,

```
ref_fa=path/to/t2t-and-subtel.fa
telogator2.py -i ${sample}_sorted.bam -o ${prefix} \
-p 24 -r ont --ref $ref_fa -n 4 --minimap2 path/to/minimap2
```

Whole genomeTCGA-OV [41] WGS data were retrieved from the GDC portal [42], and the average telomere length of all chromosomes for each sample was estimated using Telseq [43]. Samples with the name "NB" were classified as normal blood cells, "PT" as ovarian tumor tissue, and "NT" as non-cancerous tissue.

For TelSeq,
```
telseq $inBAM > $output.tsv
```

Validation of methylation profiles of the studied samples using external data

Illumina EPIC DNA methylation array data were obtained for normal white blood cells (n=54, GSE213478) from the Genotype-Tissue Expression (GTEX) [44] and for HGSOC (n=253, GSE263434) from JGOG3025-TR2 [45]. A previous study [46] provide a correspondence table linking EPIC probes to CpG sites in the T2T-CHM13 genome.

Of 735,112 annotated probes, 9,717 showed an average beta value difference of >0.5 and 18,236 a difference of <−0.5 between HGSOC and WBC. All CpG sites in these probes were converted to the T2T-CHM13 genomic coordinates using the correspondence table above, and their 5mC methylation level in LRS samples were examined (Supplementary Figure7B).

Software versions

Software	Version	URL	Reference
MinKNOW	24.02.10/24.06.10/24.06.14	https://community.nanoporetech.com/downloads	https://oxfordnanoporedx.com
Dorado	7.3.9/7.4.12/7.4.13	https://github.com/nanoporetech/dorado	https://github.com/nanoporetech
minimap2	2.28-r1209	https://github.com/lh3/minimap2	PMID: 29750242
Clair3	1.0.9	https://github.com/HKU-BAL/Clair3	PMID: 38177392
longphase	1.7.3	https://github.com/twolinin/longphase	PMID: 35104333
ClairS	0.4.0	https://github.com/HKU-BAL/ClairS	https://doi.org/10.1101/2023.08.17.553778
Ensembl-vep	111.0	https://github.com/Ensembl/ensembl-vep	PMID: 27268795
oncokb-annotator	accessed on Nov 15, 2024	https://github.com/oncokb/oncokb-annotator	PMID: 28890946
vcf2maf	1.6.21	https://github.com/mskcc/vcf2maf	https://doi.org/10.5281/zenodo.1185418
SigProfilerExtractor	1.1.25	https://github.com/AlexandrovLab/SigProfilerExtractor	PMID: 36388765
alleleCount	4.2.1	https://github.com/cancerit/alleleCount	https://doi.org/10.5281/zenodo.593129
ascatNgs	3.1.3	https://github.com/cancerit/ascatNgs	PMID: 27930809
scarHRD	No release version	https://github.com/sztup/scarHRD	PMID: 29978035
Severus	1.2	https://github.com/KolmogorovLab/Severus	https://doi.org/10.1101/2024.03.22.24304756
SAVANA	1.2.4	https://github.com/cortes-ciriano-lab/savana	https://doi.org/10.1101/2024.07.25.604944
nanomonsv	0.7.2	https://github.com/friend1ws/nanomonsv	PMID: 37336583
DELLY	1.3.1	https://github.com/dellytools/delly	PMID: 22962449
AnnotSV	3.4.4	https://github.com/lgmgeo/AnnotSV	PMID: 29669011
SURVIVOR	1.0.6	https://github.com/fritzsedlazeck/SURVIVOR	PMID: 28117401
CNVkit	0.9.9	https://github.com/etal/cnvkit	PMID: 27100738
JaBbA	1.1	https://github.com/mskilab-org/JaBbA	PMID: 33007263
mosdepth	0.3.10	https://github.com/brentp/mosdepth	PMID: 29096012
modkit	0.4.3	https://github.com/nanoporetech/modkit	https://github.com/nanoporetech
tldr	1.2.2	https://github.com/adamewing/tldr	PMID: 33186547
telogator2	No release version	https://github.com/zstephens/telogator2	PMID: 38755561
Telometer	1.0	https://github.com/santiago-es/Telometer	PMID: 38890274
telseq	0.0.2	https://github.com/zd1/telseq	PMID: 24609383

Supplementary References

1. Zheng Z, Su J, Chen L, Lee Y-L, Lam T-W, Luo R. ClairS: a deep-learning method for long-read somatic small variant calling. bioRxiv. 2023. doi:10.1101/2023.08.17.553778

2. Xu L, Liddell B, Nesic K, Geissler F, Ashwood LM, Wakefield MJ, et al. High-level tumour methylation of BRCA1 and RAD51C is required for homologous recombination deficiency in solid cancers. NAR Cancer. 2024;6: zcae033.

3. Raine KM, Van Loo P, Wedge DC, Jones D, Menzies A, Butler AP, et al. ascatNgs: Identifying Somatically Acquired Copy-Number Alterations from Whole-Genome Sequencing Data. Curr Protoc Bioinformatics. 2016;56: 15.9.1-15.9.17.

4. Shiraishi Y, Koya J, Chiba K, Okada A, Arai Y, Saito Y, et al. Precise characterization of somatic complex structural variations from tumor/control paired long-read sequencing data with nanomonsv. Nucleic Acids Res. 2023;51: e74.

5. Elrick H, Sauer CM, Espejo Valle-Inclan J, Trevers K, Tanguy M, Zumalave S, et al. SAVANA: reliable analysis of somatic structural variants and copy number aberrations using long-read sequencing. Nat Methods. 2025;22: 1436–1446.

6. Keskus A, Bryant A, Ahmad T, Yoo B, Aganezov S, Goretsky A, et al. Severus: accurate detection and characterization of somatic structural variation in tumor genomes using long reads. medRxiv. 2024. doi:10.1101/2024.03.22.24304756

7. Rausch T, Zichner T, Schlattl A, Stütz AM, Benes V, Korbel JO. DELLY: structural variant discovery by integrated paired-end and split-read analysis. Bioinformatics. 2012;28: i333–i339.

8. Geoffroy V, Herenger Y, Kress A, Stoetzel C, Piton A, Dollfus H, et al. AnnotSV: an integrated tool for structural variations annotation. Bioinformatics. 2018;34: 3572–3574.

9. Choo ZN, Behr JM, Deshpande A, Hadi K, Yao X, Tian H, et al. Most large structural variants in cancer genomes can be detected without long reads. Nat Genet. 2023;55: 2139–2148.

10. Sanchez SE, Gu Y, Wang Y, Golla A, Martin A, Shomali W, et al. Digital telomere measurement by long-read sequencing distinguishes healthy aging from disease. Nat Commun. 2024;15: 5148.

11. Stephens Z, Kocher J-P. Characterization of telomere variant repeats using long reads enables allele-specific telomere length estimation. BMC Bioinformatics. 2024;25: 194.

12. MinKNOW Software. In: MinKNOW Software downloads [Internet]. Available: https://community.nanoporetech.com/downloads

13. Dorado: Oxford nanopore's basecaller. Github; Available: https://github.com/nanoporetech/dorado

14. Li H. Minimap2: Pairwise alignment for nucleotide sequences. Bioinformatics. 2018;34: 3094–3100.

15. Zheng Z, Li S, Su J, Leung AW-S, Lam T-W, Luo R. Symphonizing pileup and full-alignment for deep learning-based long-read variant calling. Nat Comput Sci. 2022;2: 797–803.

16. Lin J-H, Chen L-C, Yu S-C, Huang Y-T. LongPhase: an ultra-fast chromosome-scale phasing algorithm for small and large variants. Bioinformatics. 2022;38: 1816–1822.

17. McLaren W, Gil L, Hunt SE, Riat HS, Ritchie GRS, Thormann A, et al. The Ensembl Variant Effect Predictor. Genome Biol. 2016;17: 122.

18. Chakravarty D, Gao J, Phillips SM, Kundra R, Zhang H, Wang J, et al. OncoKB: A precision oncology knowledge base. JCO Precis Oncol. 2017;2017. doi:10.1200/PO.17.00011

19. Ng PC, Henikoff S. SIFT: Predicting amino acid changes that affect protein function. Nucleic Acids Res. 2003;31: 3812–3814.

20. Adzhubei I, Jordan DM, Sunyaev SR. Predicting functional effect of human missense mutations using PolyPhen-2. Curr Protoc Hum Genet. 2013;Chapter 7: Unit7.20.

21. Jaganathan K, Kyriazopoulou Panagiotopoulou S, McRae JF, Darbandi SF, Knowles D, Li YI, et al. Predicting splicing from primary sequence with deep learning. Cell. 2019;176: 535-548.e24.

22. Islam SMA, Díaz-Gay M, Wu Y, Barnes M, Vangara R, Bergstrom EN, et al. Uncovering novel mutational signatures by de novo extraction with SigProfilerExtractor. Cell Genom. 2022;2: None.

23. Sondka Z, Dhir NB, Carvalho-Silva D, Jupe S, Madhumita, McLaren K, et al. COSMIC: a curated database of somatic variants and clinical data for cancer. Nucleic Acids Res. 2024;52: D1210–D1217.

24. Raine K, Jones D, AndyMenzies, Alcantara R. cancerit/alleleCount: v4.2.1: htslib 1.11+libdeflate. Zenodo; 2020. doi:10.5281/ZENODO.593129

25. ascat: ASCAT R package. Github; Available: https://github.com/VanLoo-lab/ascat

26. Sztupinszki Z, Diossy M, Krzystanek M, Reiniger L, Csabai I, Favero F, et al. Migrating the SNP array-based homologous recombination deficiency measures to next generation sequencing data of breast cancer. npj Breast Cancer. 2018;4: 8–11.

27. Liu L, Zhang J, Wood S, Newell F, Leonard C, Koufariotis LT, et al. Performance of somatic structural variant calling in lung cancer using Oxford Nanopore sequencing technology. BMC Genomics. 2024;25: 898.

28. Aydin SK, Yilmaz KC, Acar A. Benchmarking long-read structural variant calling tools and combinations for detecting somatic variants in cancer genomes. Sci Rep. 2025;15: 8707.

29. Wang M, Luo W, Jones K, Bian X, Williams R, Higson H, et al. SomaticCombiner: improving the performance of somatic variant calling based on evaluation tests and a consensus approach. Sci Rep. 2020;10: 12898.

30. Schloissnig S, Pani S, Rodriguez-Martin B, Ebler J, Hain C, Tsapalou V, et al. Long-read sequencing and structural variant characterization in 1,019 samples from the 1000 Genomes Project. bioRxiv. 2024. doi:10.1101/2024.04.18.590093

31. Jeffares DC, Jolly C, Hoti M, Speed D, Shaw L, Rallis C, et al. Transient structural variations have strong effects on quantitative traits and reproductive isolation in fission yeast. Nat Commun. 2017;8: 14061.

32. Talevich E, Shain AH, Botton T, Bastian BC. CNVkit: Genome-wide copy number detection and visualization from targeted DNA sequencing. PLoS Comput Biol. 2016;12: e1004873.

33. Gurobi Optimization, LLC. Gurobi Optimizer Reference Manual. 2024. Available: https://www.gurobi.com

34. Altemose N, Logsdon GA, Bzikadze AV, Sidhwani P, Langley SA, Caldas GV, et al. Complete genomic and epigenetic maps of human centromeres. Science. 2022;376. doi:10.1126/science.abl4178

35. Li H, Handsaker B, Wysoker A, Fennell T, Ruan J, Homer N, et al. The Sequence Alignment/Map format and SAMtools. Bioinformatics. 2009;25: 2078–2079.

36. Pedersen BS, Quinlan AR. Mosdepth: quick coverage calculation for genomes and exomes. Bioinformatics. 2018;34: 867–868.

37. Hoyt SJ, Storer JM, Hartley GA, Grady PGS, Gershman A, de Lima LG, et al. From telomere to telomere: The transcriptional and epigenetic state of human repeat elements. Science. 2022;376: eabk3112.

38. modkit: A bioinformatics tool for working with modified bases. Github; Available: https://github.com/nanoporetech/modkit

39. Quinlan AR, Hall IM. BEDTools: a flexible suite of utilities for comparing genomic features. Bioinformatics. 2010;26: 841–842.

40. Stong N, Deng Z, Gupta R, Hu S, Paul S, Weiner AK, et al. Subtelomeric CTCF and cohesin binding site organization using improved subtelomere assemblies and a novel annotation pipeline. Genome Res. 2014;24: 1039–1050.

41. Bell D, Berchuck A, Birrer M, Chien J, Cramer DW, Dao F, et al. Integrated genomic analyses of ovarian carcinoma. Nature. 2011;474: 609–615.

42. Heath AP, Ferretti V, Agrawal S, An M, Angelakos JC, Arya R, et al. The NCI genomic data commons. Nat Genet. 2021;53: 257–262.

43. Ding Z, Mangino M, Aviv A, Spector T, Durbin R, UK10K Consortium. Estimating telomere length from whole genome sequence data. Nucleic Acids Res. 2014;42: e75.

44. Oliva M, Demanelis K, Lu Y, Chernoff M, Jasmine F, Ahsan H, et al. DNA methylation QTL mapping across diverse human tissues provides molecular links between genetic variation and complex traits. Nat Genet. 2023;55: 112–122.

45. Takamatsu S, Hillman RT, Yoshihara K, Baba T, Shimada M, Yoshida H, et al. Molecular classification of ovarian high-grade serous/endometrioid carcinomas through multi-omics analysis: JGOG3025-TR2 study. Br J Cancer. 2024;131: 1340–1349.

46. Dong Z (joe), Whitehead J, Fu M, MacIsaac JL, Rehkopf DH, Rosero-Bixby L, et al. Complete reference genome and pangenome expand biologically relevant information for genome-wide DNA methylation analysis using short-read sequencing and array data. bioRxiv. 2024. doi:10.1101/2024.10.07.617116
